# Oblique and rippled heliosphere structures from the Interstellar Boundary Explorer

**DOI:** 10.1038/s41550-022-01798-6

**Published:** 2022-10-10

**Authors:** Eric J. Zirnstein, Bishwas L. Shrestha, David J. McComas, Maher A. Dayeh, Jacob Heerikhuisen, Daniel B. Reisenfeld, Justyna M. Sokół, Paweł Swaczyna

**Affiliations:** 1 Department of Astrophysical Sciences, Princeton University, Princeton, NJ USA; 2 Southwest Research Institute, San Antonio, TX USA; 3 Department of Physics and Astronomy, University of Texas at San Antonio, San Antonio, TX USA; 4 Department of Mathematics and Statistics, University of Waikato, Hamilton, New Zealand; 5 Los Alamos National Laboratory, Los Alamos, NM USA

**Keywords:** Solar physics, Interstellar medium, Astrophysical plasmas, Space physics

## Abstract

Past analysis has shown that the heliosphere structure can be deduced from correlations between long-scale solar wind pressure evolution and energetic neutral atom emissions. However, this required spatial and temporal averaging that smoothed out small or dynamic features of the heliosphere. In late 2014, the solar wind dynamic pressure increased by roughly 50% over a period of 6 months, causing a time and directional-dependent rise in around 2–6 keV energetic neutral atom fluxes from the heliosphere observed by the Interstellar Boundary Explorer. Here, we use the 2014 pressure enhancement to provide a simultaneous derivation of the three-dimensional heliospheric termination shock (HTS) and heliopause (HP) distances at high resolution from Interstellar Boundary Explorer measurements. The analysis reveals rippled HTS and HP surfaces that are oblique with respect to the local interstellar medium upwind direction, with significant asymmetries in the heliosphere structure compared to steady-state heliosphere models. We estimate that the heliosphere boundaries contain roughly ten astronomical unit-sized spatial variations, with slightly larger variations on the HTS surface than the HP and a large-scale, southwards-directed obliquity of the surfaces in the meridional plane. Comparisons of the derived HTS and HP distances with Voyager observations indicate substantial differences in the heliosphere boundaries in the northern versus southern hemispheres and their motion over time.

## Abstract (web summary)

Energetic neutral atom fluxes measured at 1 au by the IBEX spacecraft between 2014 and 2019 are used as proxy to map the heliosphere at high resolution. Persistent ripples that corrugate the heliospheric boundary and induce variations by up to ~10 au are observed, with marked north–south asymmetry.

## Main

The heliosphere surrounding our solar system is formed by the interaction between the solar wind (SW) and the partially ionized, local interstellar medium (LISM)^1^. The interstellar plasma, consisting mostly of H and He, is slowed at the bow wave upstream of the heliosphere^2,3^ and diverted around the heliopause (HP)^4–7^. Interstellar neutral atoms, however, can cross the HP and enter the heliosphere^8–10^. Low energy interstellar neutrals are detected directly by the Interstellar Boundary Explorer (IBEX)^11^ near Earth^12,13^ and Ulysses GAS^14,15^, but they also may undergo charge exchange collisions inside the heliosphere. The ionization of interstellar neutrals in the supersonic SW and inner heliosheath (IHS) produces energetic pickup ions (PUIs) that dominate the plasma pressure. Through another charge exchange collision, PUIs create energetic neutral atoms (ENAs) at energies much greater than the interstellar neutrals. IBEX measures ENA fluxes at energies up to roughly 6 keV from all directions of the sky and has accumulated more than a solar cycle of ENA observations since 2009 (ref. ^16^).

**Fig. 1:**
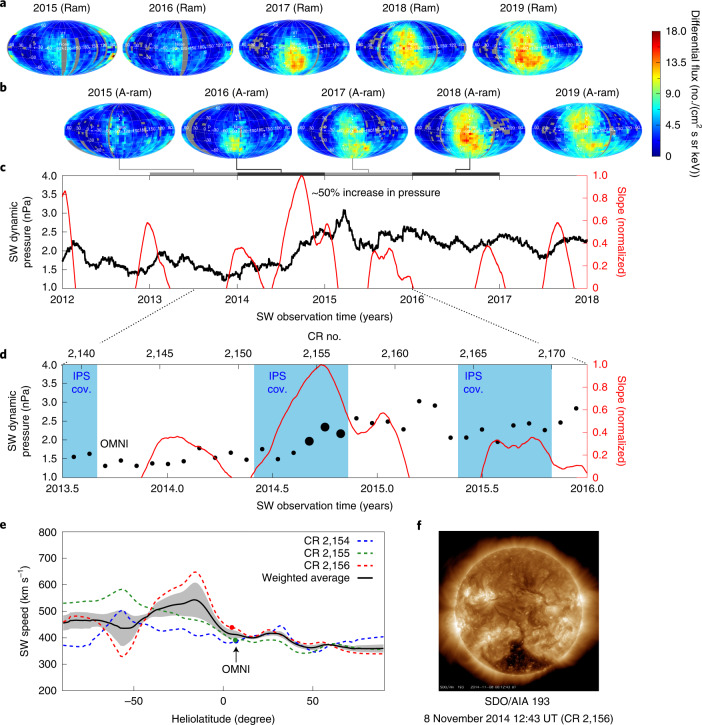
IBEX ENA fluxes and SW properties. **a**,**b**, IBEX ENA sky maps are shown for roughly 3–6 keV ENA fluxes (McComas et al.^16^). **a**, Ram maps correspond to times when observations are made in the spacecraft ram frame. **b**, Antiram (A-ram) maps are observations made in the antiram frame. Pixels in corresponding years between rows **a** and **b** are offset by 6 months. IBEX observations are corrected for the Compton–Getting effect when transforming from the spacecraft frame to the solar inertial frame and corrected for ENA losses between 100 and 1 au. **c**, SW dynamic pressure observed by ACE and Wind at 1 au in the ecliptic plane (black), smoothed over two CRs. Approximate time delays between SW and ENA observations are illustrated by the coloured grey bars. The running linear slope fit to SW pressure over ±3 CRs is shown in red. **d**, IPS observations cover the three CRs nearest to the peak change in SW dynamic pressure (CR 2,154–2,156, large black dots), centred on 2014.75. **e**, IPS-derived SW speeds as a function of heliolatitude during CR 2,154–2,156. Speeds are shifted uniformly to match OMNI at low latitudes during each CR (OMNI, coloured points). We use SW observations weight averaged over this period (black curve) to analyse IBEX observations. The grey contour represents the propagated standard deviation of the average. **f**, A large coronal hole in the southern hemisphere, visible in SDO/AIA observations as the dark colour spot, resulted in fast SW at mid-latitudes in CR 2,156 (image courtesy of NASA/SDO and the AIA science team).

It has become clear over the past decade that the heliosphere can respond globally to large-scale changes in the SW dynamic pressure. Voyager observations within the IHS have shown large variability in the magnetic field, thermal ion properties and transients propagating across the IHS and into the LISM^17–19^, as well as the dynamic characteristics of the heliosphere boundaries^5,6,20,21^, but only along their respective trajectories. IBEX, with its ability to map the entire sky every 6 months, has revealed both gradual, long-term changes in ENA fluxes^16,22,23^ and abrupt, short-term variability linked to changes in the SW dynamic pressure^24,25^. A large increase in SW dynamic pressure observed by ACE and Wind in late 2014 at 1 au (Fig. 1) was reflected in enhanced ENA emissions measured by IBEX beginning in late 2016. Increased ENA fluxes were first seen roughly 30° below the nose of the heliosphere^24^ (that is, the LISM upwind flow direction), followed by enhancements over larger regions of the sky later in time^16,25^. The spatially dependent response of heliospheric ENAs to the SW pressure change were shown to be caused by the asymmetric structure of the heliosphere^26^.

With the only two in situ measurements of the heliosphere boundaries from the Voyager spacecraft^4,6,21^, as well as determinations of the heliospheric termination shock (HTS) structure on the flanks from the Voyagers’ magnetic disconnection events in the IHS^27^, the heliospheric community has realized the importance of using ENA imaging to detect and correlate changes in the SW with ENA emissions across the sky. Reisenfeld et al. (ref. ^28^) recently demonstrated how IBEX measurements can be used to map the three-dimensional heliospheric structure on large scales using a combination of SW observations at 1 au and global simulations that inform us of the behaviour of ENA emissions in the IHS. With the intention of studying the time-averaged shape of the HP, the methods used by Reisenfeld et al. allowed for the estimation of the HP boundary at large scales over nearly the entire sky. However, the boundaries of the HTS and HP are expected to move on the order of 10 au over a solar cycle^29,30^, or perhaps more if Rayleigh–Taylor and Kelvin–Helmholtz instabilities of the HP surface are strong and prevalent^31,32^. These variations are within the uncertainties of the time-averaged model demonstrated by Reisenfeld et al. For the present study, where interests lie in understanding small-scale fluctuations in the HTS and HP surfaces, the methodology used by Reisenfeld et al. must be modified.

A substantial advancement of the current study compared to previous analyses is the determination of the HTS shape directly from IBEX observations, without assuming the HTS shape a priori from preexisting models. We use observations of a single, global increase in SW dynamic pressure in late 2014 and two separate temporal features observed in IBEX ENA fluxes as they respond to this SW pressure event between 2016 and 2019 to derive the shape of the HTS and HP over a roughly 2 year time span. This methodology allows us to derive the HTS and HP surfaces at higher resolution than previous analyses, but it can only be applied to directions in the sky where ENA emissions respond strongly to the solar event, that is, where the IHS is closest to the Sun. Thus, our analysis is confined to the half of the sky centred on the direction where ENAs first responded to the global pressure event.

We use IBEX observations of roughly 1.4–6 keV ENA fluxes from 2014 to 2019 in our analysis. As IBEX orbits around Earth, it spins along a Sun-pointed axis allowing it to map the sky every 6 months. Data are collected in the spacecraft frame of reference as the Earth orbits the Sun, both in its ‘ram’ reference frame where it is moving towards the ENA source and the ‘antiram’ reference frame where it is moving away from the ENA source^33^. The data are transformed into the solar inertial frame by correcting for the Compton–Getting effect^34,35^ and are corrected for ENA losses within roughly 100 au of the Sun^16^. Because the IBEX ribbon overlaps a substantial part of the globally distributed flux (GDF) near the upwind hemisphere^12^, most studies of the GDF require removal of the ribbon feature by using a combination of subtraction, masking, interpolation and reconstruction^28,36–38^. However, our analysis does not require removal of the ribbon because the GDF signal that is rapidly changing in response to the SW pressure increase is much stronger than the slowly varying ribbon flux behind it at the electrostatic analyser (ESA) energy steps examined here. This is primarily due to the longer line of sight (LOS) thickness of the ribbon source region outside the HP^39,40^.

The spatially dependent response of heliospheric ENAs to the SW pressure increase indicates the asymmetric structure of the heliosphere boundaries, as was demonstrated by a global magnetohydrodynamic (MHD) simulation^26^. The timing of the ENA response is correlated with the time for magnetosonic wave propagation from the HTS to the HP and approximately halfway back (that is, near the middle of the ENA source region), which was interpreted as a measure of the time it takes the IHS to respond to global changes in SW pressure. This correlation was used to estimate the distance to the HP across the entire sky, averaged over a solar cycle^28^. On further analysis of global MHD simulations, however, we find that the response of ENAs in the IHS to a strong, global pressure change such as that which occurred in late 2014 (roughly 50% increase in dynamic pressure) is driven by both the magnetosonic wave speed and the flow advection speed, and the point in time where the pressure wave reflected from the HP interacts with the higher-pressure advecting flow somewhere in the IHS.

**Fig. 2:**
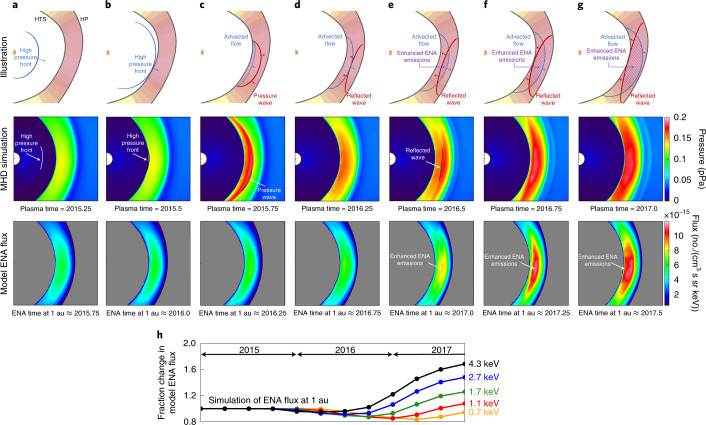
Illustration of IHS ENA response to global SW pressure increase after late 2014. **a**, SW with high dynamic pressure is emitted from the Sun in late 2014, reaching halfway to the HTS in early 2015. **b**, The SW pressure front reaches the HTS in mid-2015. **c**, After reaching the HTS, a pressure wave travels at the fast magnetosonic speed through the IHS, leading the slower, advected plasma flow. **d**, After reaching the HP, a reflected wave travels back towards the HTS and intersects with the advected flow approximately midway through the IHS. **e**, After the reflected wave and advected flow intersect, ENA emissions begin increasing within their intersected region. **f**, After travelling further across each other, ENA emissions rise substantially over a larger area. **g**, Enhanced ENA emissions have approximately filled the IHS by the time the reflected wave reaches the HTS. The second row of panels show plasma pressure from a 3D, dynamic MHD simulation of this event^31,33^. White and black contours of the wave fronts have been drawn to help guide the eye. The third row of panels show model ENA fluxes emitted in the IHS that would be observed by IBEX roughly 6 months later at roughly 4.3 keV. **h**, Fraction change in model ENA flux at 1 au from a 20° × 20° region in the sky centred near the direction of maximum emission (adapted from ref. ^31^).

This process is demonstrated in Fig. 2, which shows an illustration of the advection of the high-pressure plasma and travelling magnetosonic wave in the IHS. First, the high-pressure wave front released from the Sun in late 2014 has travelled halfway to the HTS by early 2015 (Fig. 2a) and reaches the nose-ward HTS in mid-2015 (Fig. 2b). After reaching the HTS, a pressure wave travelling at the fast magnetosonic speed is released and locally heats the thermal plasma as it travels through the IHS but does not yet noticeably increase ENA emissions. The pressure wave reaches the HP in late 2015/early 2016, after which a reflected wave travels back towards the HTS. By mid-2016, the advecting flow and reflected wave have met near the middle of the IHS and crossed each other: their interaction results in an increase in ENA emissions by adiabatic heating of the advecting plasma, which is observed roughly 6 months later at 1 au as increased intensities of roughly 4 keV ENAs. This process is demonstrated by simulations shown in Fig. 2. Assuming that the simulation can be used to predict how the heliosphere and ENA emissions qualitatively behave to a rapid increase in SW dynamic pressure, we can derive the distance to the HTS and HP directly from IBEX observations.

## Results

**Fig. 3:**
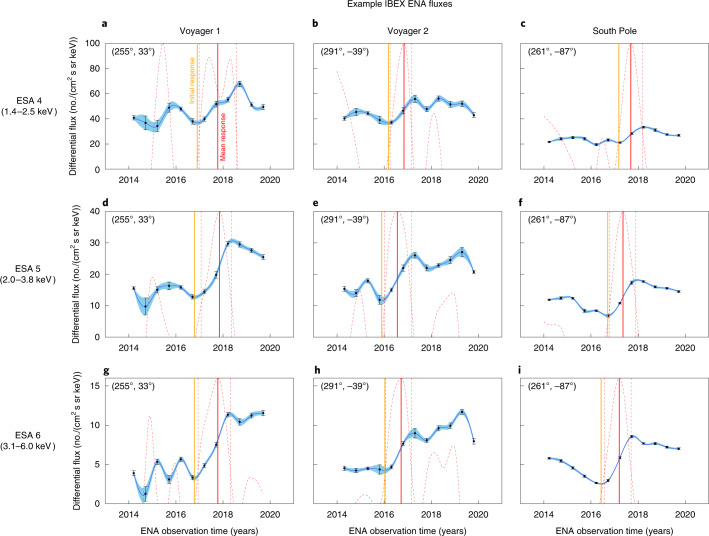
Examples of IBEX ENA time series. **a**–**i**, ENA fluxes at ESA 4 (**a**–**c**), ESA 5 (**d**–**f**) and ESA 6 (**g**–**i**) in several directions of the sky (6° × 6° bins) and their statistical uncertainties: Voyager 1 (**a**,**d**,**g**), Voyager 2 (**b**,**e**,**h**) and South Pole (**c**,**f**,**i**). A cubic spline is fit to IBEX data (blue curve with propagated uncertainty contour), and a running slope over ±0.5 years is calculated at each point of the spline (red dashed curve: note that the slope is normalized to the *y* axis range). We first determine a time range surrounding the time of maximum slope in ENA flux. This range is bounded by the times when the slope is 25% of the peak (red vertical dashed lines). The mean ENA response time is defined as the point of maximum slope within this range (red vertical solid line). However, if multiple peaks in the slope exist within this range (for example, in **a**), the mean response time is defined as the middle of the range. The initial ENA response time is found at the point of zero slope in ENA flux (orange vertical solid line) that occurs before the mean response time. Note that the uncertainties in the South Pole are smaller primarily due to the higher exposure time per pixel at the poles.

Using the fact that the SW pressure increase observed in late 2014 was probably a global event^16^, we first identify the time in IBEX observations that ENAs began responding to the SW pressure change for each pixel in the sky. We limit our analysis to pixels within 90° of the approximate direction from which the ENAs first responded (255°.7, −27°), beyond which the ENA response has either not yet been observed or is too weak to identify. Figure 3 shows examples of ENA fluxes in different directions of the sky for ESA 4–6 and the substantial rise in ENA flux in response to the SW pressure increase. The response of ENA fluxes from the heliosheath is strongest for energies roughly 3–6 keV and becomes weaker at lower energies, although still visible down at energies in ESA 4 (1.4–2.5 keV). We use cubic spline interpolation to interpolate ENA fluxes between IBEX data points with 0.01 year resolution and calculate the local linear slope of the spline over a running ±0.5 year window. The point of maximum slope signifies the time when the response of ENA fluxes to the SW pressure is changing the most rapidly. This point in time is approximately when the line-of-sight integrated ENA emissions has reached roughly 50% of its maximum, hereafter called the ‘mean ENA response time’.

### ENA response times

**Fig. 4:**
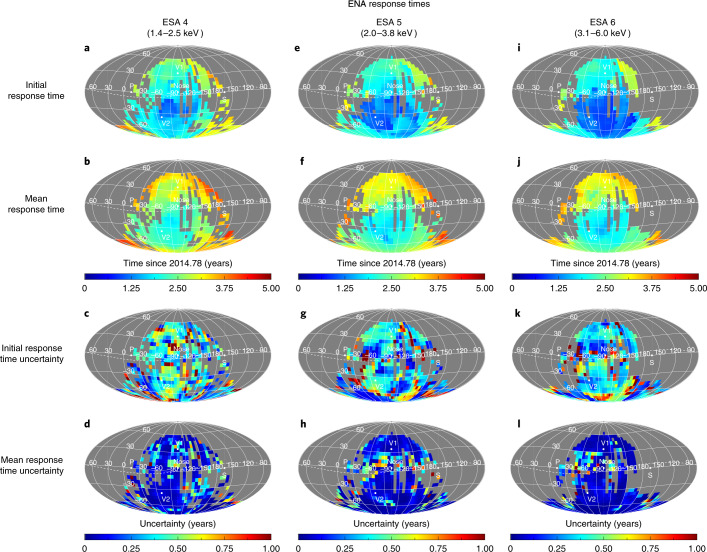
All sky maps of IBEX ENA response times. **a**–**l**, The initial (**a**,**e**,**i**) and mean (**b**,**f**,**j**) response times and their corresponding uncertainties (initial response uncertainties in panels **c**,**g**,**k** and mean response uncertainties in panels **d**,**h**,**l**) are shown for each pixel in the sky accepted in the analysis, separately for ESA 4 (**a**–**d**), ESA 5 (**e**–**h**) and ESA 6 (**i**–**l**). Uncertainties for the initial and mean ENA response times are calculated by propagating the uncertainty of each IBEX data point. An additional uncertainty in the initial response time is included by calculating the effect that fluctuations in ENA flux before the initial response time may have on the result. See Methods for more details. Source data

The moment in time before ENA fluxes first begin to rise is used to identify the inner boundary of the ENA emission region, that is, the HTS. As demonstrated in the simulation (Fig. 2), after the pressure front crosses the HTS and begins propagating through the IHS, the HTS moves outwards slightly and the line-of-sight integrated ENA flux decreases slightly right before any large increase in ENA emissions occurs. Therefore, we identify the time when the ENA flux is at a minimum just before the sharp rise as the ‘initial ENA response time’. We identify the initial and mean ENA response times for all available pixels in the sky, which represent approximately 34–37% of the full sky area depending on the ESA, as shown in Fig. 4. We note, however, that there is a potential issue in using this time as the location of the HTS. The time at which we identify the higher-plasma pressure has reached the HTS is probably after the HTS has already begun moving outwards before observing any significant increase in ENA intensity. Therefore, we must interpret this as the maximum distance to the HTS compared to its state before modification by the higher-pressure plasma. The uncertainties in the initial ENA response time are significantly larger than the mean response time due to the variability in ENA flux that occurs before the heliosphere responds to the global SW pressure event.

After identifying the initial and mean ENA response times for ESA 4–6, shown in Fig. 4, we calculate the distance to the HTS, mean ENA source and HP using the steps described in the Methods and summarized here: first, we calculate the distance to the HTS by integrating (1) the time that the SW travels from the Sun to distance *r* and (2) the time that ENAs within each ESA travel from distance *r* back to Earth, until the total time passed equals the initial ENA response time observed by IBEX. We use measurements of SW speed, density, temperature and magnetic field from the OMNI database (in-ecliptic SW)^41^ and interplanetary scintillation (IPS) (out of the ecliptic plane) measurements^42^ corresponding to the time frame of the SW pressure increase in late 2014. We solve a set of multi-fluid equations for conservation of mass, momentum and pressure of the SW protons, alphas, H^+^ PUIs and He^+^ PUIs, with source terms for interstellar H and He neutrals ionized by charge exchange and photoionization^43–45^, separately deriving distances to the HTS, *r*_HTS_, for each pixel. The calculation is weighted by the product of the instrument response function^46^ and ENA source spectrum derived from the GDF^38^ for ESA 4–6.

### Heliosphere boundary distances

**Fig. 5:**
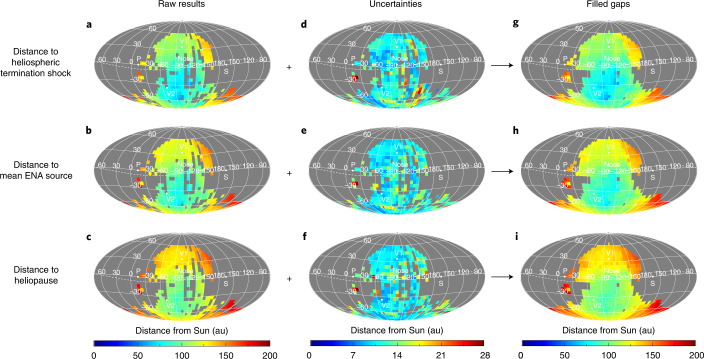
Energy-combined sky maps of distances to the HTS, mean ENA source in the IHS and HP. **a**–**c**, Distances to the heliosphere boundaries are calculated from the ENA measurement times in Fig. 4 and weight averaged over IBEX energy passbands 4–6: to heliosphere termination shock (**a**), to mean ENA source (**b**) and to HP (**c**). **d**–**f**, Propagated uncertainties of the raw distance results: to HTS (**d**), to mean ENA source (**e**) and to HP (**f**). **g**–**i**, A 10° statistical smoothing is performed to fill in gaps for illustrative purposes: to HTS (**g**), to mean ENA source (**h**) and to HP (**i**). A minimum of three nearby pixels within 10° (measured from the pixel centres) is required for filling; otherwise, the data is not modified and existing data are not changed. Source data

The distances to the mean ENA source, *r*_ENA_, and HP, *r*_HP_, are calculated simultaneously using the difference in time between the initial ENA response time, *t*_HTS_, and the mean ENA response time, *t*_ENA_, which is the time it takes for the advecting high-pressure plasma to cross the reflected wave over enough distance that the line-of-sight integrated ENA flux reaches half the final pressure state, as illustrated in Fig. 2. All flow advection and wave speeds are calculated from the coupled multi-fluid transport equations, which are advected across the HTS using the single-fluid shock adiabatic equation. Figure 5 shows the results of our analysis, after culling pixels where the ENA fluxes did not show clear behaviour related to the SW event, the uncertainties were too high or there were data gaps. Uncertainties in the distances are calculated by propagating the uncertainties of multiple variables through the analysis. Detailed descriptions of the distance derivation, data culling and uncertainty propagation procedures are provided in the Methods section.

**Fig. 6:**
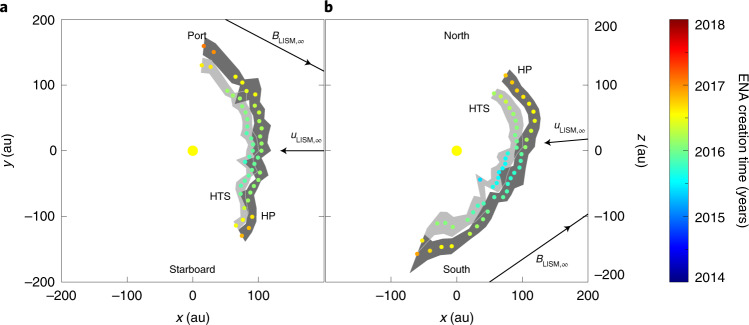
Cross sections of the heliosphere boundary distances. **a**, Cross section of distances in the ecliptic plane. **b**, Cross section of distances in the meridian plane. Distances and their uncertainties (grey contours) are derived from the maps in Fig. 5a–f. The distances to the HTS and its uncertainty range are shown as coloured dots in the light grey contour closest to the Sun, and distances to the HP and its uncertainty range are shown as coloured dots in the dark grey contour farthest from the Sun. The colours correspond to the time when the ENAs were created at that position. Projections of the LISM flow and magnetic field vectors far from the heliosphere are also shown.

Similar to previous analyses^28,47^, the closest positions of the heliosphere boundaries are not centred on the LISM upwind direction but rather a few tens of degrees below the nose of the heliosphere (Fig. 5g–i). Cross sections in the ecliptic and meridian planes (Fig. 6) show the boundary surfaces are highly oblique in the meridian plane with respect to the nose, tilted roughly 30° southwards from the ecliptic plane. There is evidence for spatial variations of the boundary distances over angular scales roughly 10° and larger, as is evident in the ‘wavy’ structure of the surfaces between neighbouring pixels. We estimate the significance of these variations by performing a minimized fit of a quadratic polynomial to the surfaces in the ecliptic plane (both *xy* and radius–longitude (*r*–*ϕ*)) and meridian plane (both *xz* and radius–latitude (*r*–*θ*)) and find a statistically significant standard deviation ranging between roughly 3 and 10 au in the ecliptic plane and roughly 5–16 au in the meridian plane (ranges indicate ±1-sigma uncertainty ranges). While it appears that there are larger variations in the HTS surface within these planes, our analysis cannot determine them to be statistically significant compared to variations in the HP surface. Spatial variations in the heliosphere boundary distances on the order of 10 au are probably too large to be caused purely by differences in measurement time of ≲1 years; therefore, they may be signatures of persistent ripples or fluctuations along the heliosphere boundary surfaces.

### Comparisons with Voyager

Table 1 shows examples of distances to the heliosphere boundaries in several directions of the sky, with comparisons to Voyager observations. Voyager 1 and 2 measurements of the HTS distance from the Sun, although separated in time by roughly 3 years, show an asymmetry of roughly 10 au. Distances derived from IBEX observations taken approximately 10 years later show a larger asymmetry of roughly 25 au, but with a large uncertainty of roughly 17 au. IBEX measurements of the distance to the HTS in the Voyager 1 and 2 directions are separated in time by roughly 0.5 years, but it is unlikely that the asymmetry reported here can be explained by motions over less than 1 year. The observed asymmetry may potentially be linked to (1) north–south asymmetries in the SW mass flux^48^, where SOHO/SWAN observations of back-scattered Lyman-α radiation suggest the existence of higher SW mass flux and/or dynamic pressure in the northern hemisphere in 2014 compared to the southern hemisphere, which might create an asymmetric heliosphere shape, in contrast to the SW mass flux observed in late 2003 relevant to Voyager 1’s HTS crossing^48^, or (2) the pressure exerted by the interstellar magnetic field on the southern hemisphere of the heliosphere^49–51^. Global, three-dimensional models of the SW-LISM interaction with dynamic SW boundary conditions have suggested that substantial distortions of the HTS surface might occur over the course of a solar cycle^30,32,51–54^, but the large asymmetries reported here, if statistically significant, have yet to be reproduced by any model.

**Table 1:** Examples of distances to the heliosphere boundaries derived from the analysis

	*r*_HTS_^a^ (au)	*σ*_*r*_ (au)	Time of creation^b^ (years)	*r*_ENA_^a^ (au)	*σ*_*r*_ (au)	Time of creation^b^ (years)	*r*_HP_^a^ (au)	*σ*_*r*_ (au)	Time of creation^b^ (years)	*l*_IHS_^a^ (au)	*σ*_*l*_, *s*_*l*_ (au)
**IBEX**											
Nose	91	9	2015.9	98	12	2016.3	105	15	2016.1	13	6
Voyager 1	103	13	2016.1	117	10	2017.0	131	9	2016.6	29	10
Voyager 2	77	11	2015.6	90	10	2016.2	103	8	2015.9	25	9
South Pole	110	9	2016.0	122	7	2016.8	135	6	2016.4	22	4
**Voyager 1**	94.0	-	2004.96	-	-	-	121.6	-	2012.65	27.6	-
**Voyager 2**	83.7	-	2007.66	-	-	-	119.0	-	2018.85	35.3	-

Notes.

^a^ Distances *r*_HTS_, *r*_ENA_, *r*_HP_ and *l*_IHS_ are derived from the raw results maps in Fig. 5a–c. *l*_IHS_ is the thickness of the IHS calculated as the difference between *r*_HP_ and *r*_HTS_. Uncertainties (*σ*_*r*_) are derived from uncertainty maps in Fig. 5d–f. Note that results near the South Pole are weight averaged over four pixels surrounding the South Pole centred at latitude −87° and longitudes 75°, 165°, 255° and 345°.

^b^ Time of creation for IBEX-derived results refers to the point in time that ENAs were created at that position, which is earlier in time than the ENA observation at 1 au. Times are calculated using the same weighted average method as for the distances. Uncertainties are typically 0.1 years.

## Discussion

The distances to the HP in the Voyager directions as observed by IBEX are intriguing and potentially controversial. The analysis suggests that the distance to the HP in the Voyager 1 direction is *r*_HP_ = 131 ± 9 au as observed in 2016.6. This result, while appearing farther than 122 au where Voyager 1 crossed the HP, is still consistent with the fact that Voyager 1 crossed the HP in late 2012 and remained outside ever since^4,5^. In 2016.6, Voyager 1 was 136 au from the Sun, and therefore slightly outside the HP derived from IBEX observations. This suggests an increase in the distance to the HP in the few years after Voyager 1 crossed into interstellar space. We also note that Reisenfeld et al. (ref. ^28^) derived a similar distance to the HP near Voyager 1, although using temporal correlations over a solar cycle, indicating that their result was largely driven by the 2014 SW pressure event.

The distance to the HP in the Voyager 2 direction derived from our analysis is *r*_HP_ = 103 ± 8 au as observed in 2015.9. At this time, Voyager 2 was 109 au from the Sun and it did not cross the HP until late 2018 at a distance of 119 au (refs. ^6,7,55^). Our results are consistent with Voyager 2 measurements within 1-sigma uncertainty. However, if the HP was as close as roughly 111 au from the Sun in 2015.9, then the HP must have then moved outwards after 2015.9 before Voyager 2 crossed it in 2018.85. Dynamic heliosphere simulations qualitatively show this outward-moving behaviour of the HP^51,56^, although we must point out that nearly all models have difficulty reproducing Voyager measurements quantitatively. We note, however, that while we have attempted to include all known uncertainties in our analysis, such as the SW speed uncertainty (Methods), potential unquantified variables may contribute to these results.

**Fig. 7:**
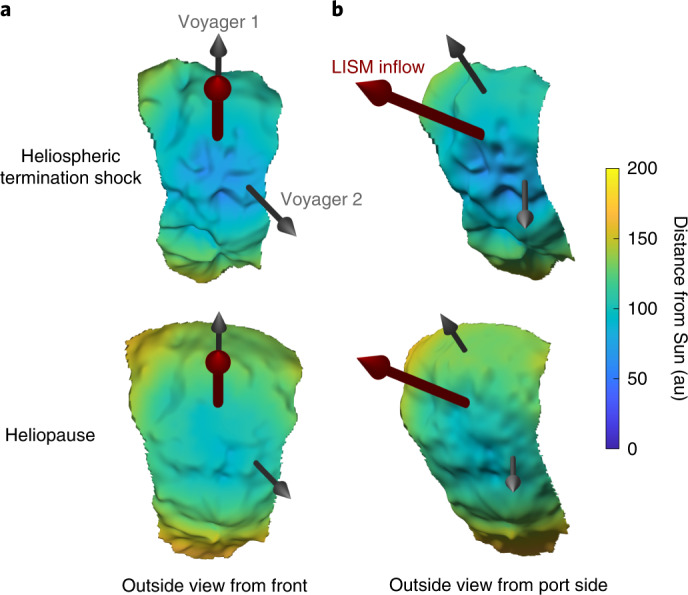
Three-dimensional visualization of the HTS and HP surfaces. **a**,**b**, Surfaces of the termination shock and HP as viewed from outside the front/upwind direction (**a**) and the port side (**b**). The surface plots are derived from the sky maps shown in Fig. 5g,i, but smoothed and interpolated to a 2° × 2° resolution grid using Kriging interpolation. Only pixels within 54° of the 255° meridian line are shown. Arrows in the direction of Voyager 1 (top grey), Voyager 2 (bottom grey) and LISM inflow directions (red) are also shown.

IBEX has operated successfully and made numerous discoveries over the past 13 years. Using IBEX observations, this study provides high-resolution maps of the heliosphere’s HTS and HP surfaces and their spatial variations (Fig. 7). While it is expected that IBEX will continue operating and taking measurements for the near future, a new NASA mission planned for launch in 2025, called the Interstellar Mapping and Acceleration Probe (IMAP)^57^, will improve on IBEX’s capabilities by measuring ENA fluxes over a larger energy range with greater accuracy and temporal resolution. IMAP is equipped with three neutral atom imagers, IMAP-Lo, IMAP-Hi and IMAP-Ultra, which will measure neutral atom fluxes from 0.005 to 1 keV, 0.4 to 16 keV and 3 to 300 keV, respectively. With their greater sensitivity, the IMAP ENA imagers will be able to produce full sky maps every 6 months and partial sky maps every 3 months, allowing us to quantify variability in the outer heliosphere at twice the cadence of IBEX. Moreover, IMAP will orbit around L1 and thus not be affected by Earth’s magnetosphere. Finally, uncertainties in the SW speed as a function of latitude (for example, discrepancies between IPS-derived speeds and the in-ecliptic measurements, or the enhanced SW flux observed by SOHO/SWAN but not by Ulysses) can be addressed with the observations of the heliospheric resonant back-scatter glow of hydrogen by IMAP-GLOWS. With these enhanced capabilities, and better understanding and removal of backgrounds, imaging the global heliosphere boundaries on the basis of the methodologies presented in this study may reveal the highly structured heliosphere evolving over time.

## Methods

### Data selection and initial processing

We analyse IBEX-Hi observations of ENAs measured within ESA energy passbands 4–6 (with a full-width at half-maximum from 1.4–2.5, 2.0–3.8 and 3.1 to 6.0 keV, respectively) starting from 2014 as part of data release 16 (ref. ^16^). ENAs measured in ESA passbands 4–6 have the highest signal to noise ratio due to the high rate of transmission through the instrument compared to lower energy passbands^46^. These ENA fluxes also show the quickest and strongest responses to the SW pressure increase, which was less noticeable at energies ≲2 keV (refs. ^24,25^). While ESA 4 fluxes show the weakest response to the SW pressure event (Fig. 3), our analysis accounts for this by yielding higher uncertainties in identifying the timing of the event (Fig. 4). ENA fluxes are observed in the spacecraft ‘ram’ frame (as IBEX is moving towards its look direction) and ‘antiram’ frame (as IBEX is moving away from its look direction), covering the sky every 6 months. We use data transformed into the solar inertial frame and corrected for ENA losses between 1 and 100 au, which removes effects of losses due to ionization close to the Sun. Each pixel in the sky has a unique time of observation as Earth orbits around the Sun each year, where IBEX starts taking observations for each ram map in the beginning of each year near 180° longitude and each antiram map starts near 0° longitude. IBEX fills in the sky over time with increasing longitude over the course of 6 months.

Before analysis, we apply a smoothing to each pixel by calculating the statistically weighted average of all pixels within 9° and applying the average to the centre pixel. This process smooths fluctuations between closely neighbouring pixels that may be a by-product of imperfect background subtraction that is performed independently for each IBEX orbital swath^33,58,59^. The smoothing also improves the capability of our analysis on deriving time delays from IBEX observations. Note, however, that because the pixels near the poles have smaller solid angle areas, spatial smoothing will inherently combine more pixels that are observed at substantially different times throughout the year. Therefore, we limit the spatial average to pixels within 9° that have measurement times within 0.25 years of the centre pixel. Because IBEX constructs all sky maps over a period of 6 months, the front half of the sky for ram measurements is constructed over the first half of the year and the back half of the sky is constructed over the second half. Because of this, data on either side of ecliptic longitude roughly 180° in ram maps are separated by 1 year in time, and data on either side of ecliptic longitude roughly 0° in antiram maps are separate by 1 year in time. Therefore, smoothing is not applied across ecliptic longitudes 180° and 0° for ram and antiram data, respectively.

Next, we apply an initial culling of the data before our analysis. First, we remove all pixels more than 90° from (255°, −27°), which is the approximate location in the sky when heliospheric ENAs first responded to the late-2014 SW pressure increase^24^. We only analyse pixels in this half of the sky because, for most observations outside this region, there has not yet been a substantial response in ENA flux to the SW pressure increase and therefore making the derivation of heliospheric distances not currently possible. Second, we remove any pixels where there are data gaps at any point in 2014–2019. After this culling, 877 of pixels in the sky remain (or roughly 48.1% of the area of the sky) out of a possible total of 1,800. We note that certain sections of the sky may have culled pixels next to unculled pixels. For example, there is a patch of culled pixels near Voyager 2 (Fig. 5a–c) that indicate potential issues in the data near that region of the sky. Therefore, extra care should be taken when interpreting results from these regions.

### Calculation of initial ENA and mean ENA responses

ENA fluxes from the outer heliosphere respond to the large increase in SW dynamic pressure a few years after in-ecliptic spacecraft first observed the SW pressure increase in late 2014. The ENA response is identified by an increase in ENA flux occurring over roughly 1–2 years. Since heliospheric ENAs cannot originate closer than the HTS, the initial rise in 3–6 keV ENA fluxes is used to identify the time at which ENAs first reacted to the SW pressure increase as it crossed the HTS. As the ENA flux continues to rise over time, the rate of increase maximizes and then gradually stops increasing. We identify the middle of this time period as the mean of the ENA source region in the IHS, as described below.

The ‘initial ENA response’ (hereafter referred to as *t*_HTS_) and ‘mean ENA response’ times (*t*_ENA_) are identified first by performing a cubic spline interpolation of the ENA flux in each non-culled pixel after 2014 with a high temporal resolution (see examples in Fig. 3). The uncertainty of the spline interpolation is calculated by propagating the data uncertainties (Calculation and propagation of uncertainties section). In the next step, the local linear slope of the spline interpolation is calculated by fitting a line to the spline and uncertainties using least-squares minimization over a ±0.5 year window. A 1-year-wide window is chosen since it represents the time over which IBEX makes at least three observations. This yields the local slope of the interpolated ENA fluxes, as shown by the red dashed curves in Fig. 3. We then find the times at which the slope reaches 25% of the peak slope in this time period (red dashed vertical lines in Fig. 3) and find the time at which the local slope is maximum, which we determine to be the ‘mean ENA response time’ (red solid vertical lines in Fig. 3). If there are multiple, large peaks in the local slope within this range, then the middle of the range is chosen as the mean response time (for example, Fig. 3a). We note that our choice of using a cubic spline to fit to IBEX data is arbitrary, and points of local maxima or minima in the slopes may shift if a different functional form was used. A higher temporal cadence of measurements from IMAP^57^ may be necessary to better constrain the appropriate fitting function.

The ‘initial ENA response’ time *t*_HTS_ is determined to be the point of local minimum in ENA flux before the mean ENA response time, which we argue is an indication of the time at which the SW pressure increase had reached the HTS and began propagating through the IHS. However, there is a degree of uncertainty as to whether the time at which the local minimum in ENA flux is truly the location of the HTS. The first reason for this is the suggestion from simulations that, as the SW pressure increase reaches the HTS, the HTS first begins to move away from the Sun as the plasma with increased pressure begins propagating through the IHS. As shown in Fig. 3 of McComas et al. (ref. ^24^), a simulation of the response of ENAs from the IHS to the SW dynamic pressure increase first resulted in a slight decrease in ENA flux before a rise in ENA flux began. This decrease appears to be a response to the outwards motion of the HTS due to the increase in SW pressure, which initially decreases the LOS-integrated ENA flux. The outwards motion of the HTS before the rise of ENA flux at 1 au is observed represents a potential uncertainty in the location of the HTS. The second reason for potential uncertainty is the existence of strong fluctuations in ENA flux observed before the rapid rise, which is evident in some pixels near the nose of the heliosphere (Fig. 3a,d,e,g). These fluctuations may adversely affect our ability in finding the ‘true’ minimum in ENA flux before the rapid rise occurs. A description for how we include these uncertainties in our analysis is given in the section Calculation and propagation of uncertainties.

After performing our analysis, a culling is applied to the results using several criteria. Results are removed if (1) the final mean ENA response time is determined to be after 2019 where we do not have enough data to confidently determine that the ENA fluxes have stopped increasing (5, 1 and 2 pixels for ESA 4, 5 and 6); (2) there are three or more peaks in the slope with heights >50% of the peak slope, making it difficult to identify the actual mean ENA response time (4, 11 and 30 pixels for ESA 4, 5 and 6); (3) the propagated uncertainty of the mean ENA response time is >1 year (76, 26 and 8 pixels for ESA 4, 5 and 6); and (4) finally, some pixels are manually removed due to complexities in the observations making it difficult to determine the ENA response times, for example, multiple peaks are visible similar to criterion no. 2, or there is no clear step function-like rise of the ENA flux (119, 111 and 145 pixels for ESA 4, 5 and 6). After final culling, 673, 728 and 692 pixels in the sky remains for ESA 4, 5 and 6, respectively.

### Calculation of distances to the HTS, mean ENA source and HP

After deriving the initial ENA response time (*t*_HTS_) and mean ENA response time (*t*_ENA_) for each accepted pixel in the sky and each ESA 4–6, we calculate the distances to the HTS, mean ENA source region and HP for each pixel. First, the distance to the HTS, *r*_HTS_, is calculated by integrating the time for SW propagation from *r*_0_ = 1 au to distance *r*, plus the time for ENA propagation from *r* back to *r*_0_, until it yields the observed initial ENA response time *t*_HTS_, such that

1 $$t_{{\mathrm{HTS}}}\left( {v_{{\mathrm{ENA}}}} \right) = \mathop {\smallint }\limits_{r_0}^{r_{{\mathrm{HTS}}}} \left( {\frac{1}{{u_{{\mathrm{SW}}}\left( {r\prime } \right)}} + \frac{1}{{v_{{\mathrm{ENA}}}}}} \right){\mathrm{d}}r\prime ,$$

where *u*_SW_ is the SW speed and *v*_ENA_ is the ENA speed. The SW speed is solved as a function of distance from the Sun using spherically symmetric, steady-state fluid transport equations for mass, momentum, magnetic field (*B*) and pressure (*p*) of the SW proton (‘SWH^+^’), alpha (‘SWHe^++^’), H^+^ PUI (‘PUIH^+^’) and He^+^ PUI (‘PUIHe^+^’) mixture with photoionization and charge exchange source terms, given as^43–45,60–62^

2 $$\frac{1}{{r^2}}\frac{{\mathrm{d}}}{{{\mathrm{d}}r}}\left( {r^2\rho u} \right) = \mathop {\sum }\limits_{i = 1}^4 S_i^\rho ,$$

3 $$\frac{1}{{r^2}}\frac{{\mathrm{d}}}{{{\mathrm{d}}r}}\left( {r^2\rho _iu} \right) = S_i^\rho ,$$

4 $$\frac{1}{{r^2}}\frac{{\mathrm{d}}}{{{\mathrm{d}}r}}\left( {r^2\rho u^2} \right) = S^m - \frac{{{\mathrm{d}}p}}{{{\mathrm{d}}r}} - \frac{{B^2}}{{\mu _0r}} - \frac{B}{{\mu _0}}\frac{{{\mathrm{d}}B}}{{{\mathrm{d}}r}},$$

5 $$u\frac{{{\mathrm{d}}p_i}}{{{\mathrm{d}}r}} = S_i^p - \gamma p_i\frac{1}{{r^2}}\frac{{\mathrm{d}}}{{{\mathrm{d}}r}}\left( {r^2u} \right),$$

6 $$\frac{1}{r}\frac{{\mathrm{d}}}{{{\mathrm{d}}r}}\left( {rBu} \right) = 0,$$

where subscript *i* represents different ion species (that is, mass and pressure terms in equations (3) and (5)). The mass source terms are given as

7 $$\begin{array}{*{20}{l}} {S_i^\rho } \hfill & = \hfill & {\left\{ {\begin{array}{*{20}{l}} {S_{{\mathrm{SWH}}^ + }^\rho = - S_{{\mathrm{cx}},{\mathrm{SWH}}^ + /{\mathrm{H}}}^\rho } \hfill \\ {S_{{\mathrm{SWHe}}^{ + + }}^\rho = 0} \hfill \\ {S_{{\mathrm{PUIH}}^ + }^\rho = S_{{\mathrm{cx}},{\mathrm{SWH}}^ + /{\mathrm{H}}}^\rho + S_{{\mathrm{ph}},{\mathrm{H}}}^\rho } \hfill \\ {S_{{\mathrm{PUIHe}}^ + }^\rho = S_{{\mathrm{ph}},{\mathrm{He}}}^\rho } \hfill \end{array},} \right.} \hfill \\ {S_{{\mathrm{cx}},i/n}^\rho } \hfill & = \hfill & {n_n\left( r \right)\sigma _{{\mathrm{ex}},i - n}\rho _iu_{{\mathrm{rel}},i - n}^\rho ,} \hfill \\ {S_{{\mathrm{ph}},n}^\rho } \hfill & = \hfill & {m_n\left( {\frac{{r_0}}{r}} \right)^2\nu _nn_n\left( r \right),} \hfill \end{array}$$

where *n* represents different neutral species. The momentum source terms are

8 $$\begin{array}{*{20}{l}} {S^m} \hfill & = \hfill & {\mathop {\sum }\limits_{i = 1}^2 \left( {S_{{\mathrm{cx}},i/n}^m - S_{{\mathrm{cx}},n/i}^m} \right) + \mathop {\sum }\limits_{n = 1}^2 \left( {S_{{\mathrm{ph}},n}^m - S_{n,{\mathrm{ph}}}^m} \right),} \hfill \\ {S_{{\mathrm{cx}},i/n}^m} \hfill & = \hfill & {n_n\left( r \right)\sigma _{{\mathrm{ex}},i - n}\rho _i\left( {u_{{\mathrm{rel}},i - n}^\rho {{{{{\mathbf{u}}}}}}_n \cdot {{{{{\mathbf{\hat r}}}}}} - \frac{{v_{th,n}^2}}{{u_{{\mathrm{rel}},i - n}^m}}\left( {{{{{{\mathbf{u}}}}}} - {{{{{\mathbf{u}}}}}}_n} \right) \cdot {{{{{\mathbf{\hat r}}}}}}} \right),} \hfill \\ {S_{{\mathrm{cx}},n/i}^m} \hfill & = \hfill & {n_n\left( r \right)\sigma _{{\mathrm{ex}},n - i}\rho _i\left( {u_{{\mathrm{rel}},n - i}^\rho u + \frac{{v_{th,i}^2}}{{u_{{\mathrm{rel}},n - i}^m}}\left( {{{{{{\mathbf{u}}}}}} - {{{{{\mathbf{u}}}}}}_n} \right) \cdot {{{{\mathbf{\hat r}}}}}} \right),} \hfill \\ {S_{{\mathrm{ph}},n}^m} \hfill & = \hfill & {m_n\left( {\frac{{r_0}}{r}} \right)^2\nu _nn_n\left( r \right){{{{{\mathbf{u}}}}}}_n \cdot {{{{{\mathbf{\hat r}}}}}},} \hfill \\ {S_{n,{\mathrm{ph}}}^m} \hfill & = \hfill & {m_n\left( {\frac{{r_0}}{r}} \right)^2\nu _nn_n\left( r \right)u,} \hfill \end{array}$$

where the first summation over ions *i* is for charge exchange between SW protons (*i* = 1) or H^+^ PUIs (*i* = 2) and neutral H, and the second summation over neutrals *n* is for photoionization of interstellar H (*n* = 1) and He (*n* = 2). The pressure source terms are

9 $$\begin{array}{*{20}{l}} {S_i^P} \hfill & = \hfill & {\left\{ {\begin{array}{*{20}{l}} {S_{{\mathrm{SWH}}^ + }^P = - S_{{\mathrm{cx}},{\mathrm{H}}/{\mathrm{SWH}} + }^p} \hfill \\ {S_{{\mathrm{SWHe}}^{ + + }}^P = 0} \hfill \\ {S_{{\mathrm{PUIH}}^ + }^P = S_{{\mathrm{cx}},{\mathrm{SWH}} + /{\mathrm{H}}}^p + S_{{\mathrm{cx}},{\mathrm{PUIH}} + /{\mathrm{H}}}^p - S_{{\mathrm{cx}},{\mathrm{H}}/{\mathrm{PUIH}} + }^p + S_{{\mathrm{ph}},{\mathrm{H}}}^p} \hfill \\ {S_{{\mathrm{PUI}},{\mathrm{He}}^ + }^P = S_{{\mathrm{ph}},{\mathrm{He}}}^p} \hfill \end{array}} \right.,} \hfill \\ {S_{{\mathrm{cx}},i/n}^p} \hfill & = \hfill & n_n\left( r \right)\sigma _{{\mathrm{ex}},i - n}\rho _i\left( {\gamma - 1} \right)\, \\&&\times \left[ {\frac{1}{2}u_{{\mathrm{rel}},i - n}^\rho \left( {{{{{{\mathbf{u}}}}}} - {{{{{\mathbf{u}}}}}}_n} \right)^2 + \frac{{v_{{\mathrm{th}},n}^2}}{{u_{{\mathrm{rel}},i - n}^m}}\left( {{{{{{\mathbf{u}}}}}} - {{{{{\mathbf{u}}}}}}_n} \right)^2 + \frac{3}{4}v_{{\mathrm{th}},n}^2u_{{\mathrm{rel}},i - n}^e} \right], \hfill \\ {S_{{\mathrm{cx}},n/i}^p} \hfill & = \hfill & {n_n\left( r \right)\sigma _{{\mathrm{ex}},n - i}\rho _i\left( {\gamma - 1} \right)\frac{3}{4}v_{{\mathrm{th}},i}^2u_{{\mathrm{rel}},n - i}^e,} \hfill \\ {S_{{\mathrm{ph}},n}^p} \hfill & = \hfill & {m_n\left( {\frac{{r_0}}{r}} \right)^2\nu _nn_n\left( r \right)\left( {\gamma - 1} \right)\left[ {\frac{1}{2}\left( {{{{{{\mathbf{u}}}}}} - {{{{{\mathbf{u}}}}}}_n} \right)^2 + \frac{3}{4}v_{{\mathrm{th}},n}^2} \right].} \hfill \end{array}$$

Since the probability of neutralization of SW alphas, which is dominated by double charge exchange with interstellar He, results in <1% loss in mass over roughly 100 au (ref. ^63^), their contribution to the momentum and pressure of the plasma mixture is negligible and thus their source terms are ignored.

The relative speeds for charge exchange in the mass, momentum and pressure source terms are given as^61^

10 $$\begin{array}{*{20}{l}} {u_{{\mathrm{rel}},i - n}^\rho = u_{{\mathrm{rel}},n - i}^\rho } \hfill & = \hfill & {\sqrt {\frac{4}{\pi }\left( {v_{{\mathrm{th}},i}^2 + v_{{\mathrm{th}},n}^2} \right) + \left( {{{{{{\mathbf{u}}}}}} - {{{{{\mathbf{u}}}}}}_n} \right)^2,} } \hfill \\ {u_{{\mathrm{rel}},i - n}^m} \hfill & = \hfill & {\sqrt {\frac{{16}}{\pi }v_{{\mathrm{th}},i}^2 + \frac{{9\pi }}{4}v_{{\mathrm{th}},n}^2 + 4\left( {{{{{{\mathbf{u}}}}}} - {{{{{\mathbf{u}}}}}}_n} \right)^2} ,} \hfill \\ {u_{{\mathrm{rel}},i - n}^e} \hfill & = \hfill & {\sqrt {\frac{4}{\pi }v_{{\mathrm{th}},i}^2 + \frac{{64}}{{9\pi }}v_{{\mathrm{th}},n}^2 + \left( {{{{{{\mathbf{u}}}}}} - {{{{{\mathbf{u}}}}}}_n} \right)^2} ,} \hfill \end{array}$$

and the charge exchange cross section for H–H^+^ is^64^

11 $$\begin{array}{l}\sigma _{{\mathrm{ex}},i - n} = \sigma _{{\mathrm{ex}},n - i} = \left[ {4.15 - 0.531{\mathrm{ln}}\left( {E_{{\mathrm{rel}},i - n}} \right)} \right]^2\left[ {1 - {\mathrm{exp}}\left( { - 67.3/E_{{\mathrm{rel}},i - n}} \right)} \right]^{4.5},\\ \quad \quad \quad \quad \quad \quad \quad E_{{\mathrm{rel}},i - n} = \frac{1}{2}m_n\left( {u_{{\mathrm{rel}},i - n}^\rho } \right)^2.\end{array}$$

Note that the gain/loss of He^+^ by charge exchange for He–He^+^ and H–He^+^ are substantially smaller than photoionization of He and can be ignored.

The total mass density is $\rho = m_{\mathrm{H}}n_{{\mathrm{SWH}}^ + } + m_{{\mathrm{He}}}n_{{\mathrm{SWHe}}^{ + + }} +$ $m_{\mathrm{H}}n_{{\mathrm{PUIH}}^ + } + m_{{\mathrm{He}}}n_{{\mathrm{PUIHe}}^ + }$, where we assume all ions co-move at bulk SW speed *u*, *γ* = 5/3 is the adiabatic index, *p*_*i*_ is the thermal pressure of each ion species, and $\nu _{\mathrm{H}} = 1.44 \times 10^{ - 7}\,{\mathrm{s}}^{ - 1}$ and $\nu _{{\mathrm{He}}} = 1.14 \times 10^{ - 7}\,{\mathrm{s}}^{ - 1}$ are the neutral H and He photoionization rates at 1 au, respectively, during Carrington rotations (CRs) 2,154–2,156 (ref. ^65^) that are varied with latitude following Bzowski et al. (ref. ^66^). Initial conditions for the SW near the ecliptic are derived from OMNI in-ecliptic observations averaged over the time of the SW pressure increase (CR 2,154–2,156). PUI H^+^ and He^+^ densities are initially zero at *r*_0_. The SW speed and density at higher latitudes *θ* are extracted from IPS observations during CR 2,154–2,156 (ref. ^42^). IPS-based SW speeds are first derived from electron density fluctuations along lines of sight near the Sun by defining a power law relationship between those density fluctuations Δ*n* and SW speed *u*, such that Δ*n* ∝ *u*^*a*^. The power law slope *a* is approximated by comparing with in-ecliptic SW measurements from the OMNI database and Ulysses observations at high latitudes. Between 1985 and 2008, a value of *a* = −0.5 was found to derive speeds that best matched OMNI and Ulysses measurements. After 2008, however, a larger, positive slope value of *a* = 1.0 was required. Tokumaru et al. (ref. ^42^) concluded that the reason for this difference is probably changes in the relationship between the density fluctuations and SW speed with different solar cycles (see their study for more details). However, while their derived SW speeds matched better to OMNI using a power law slope of *a* = 1.0, it was still overestimating OMNI SW measurements, particularly in 2014. Therefore, we shift the published IPS-derived SW speeds down by 85, 61 and 70 km s^−1^ for CR 2,154, 2,155 and 2,156, respectively, to better match OMNI. Considering that the reason for this shift is not well understood, we include a 15% relative uncertainty of the SW speeds in our analysis. After shifting the SW speeds, the plasma density as a function of latitude is calculated assuming constant dynamic pressure with latitude, that is, $\left[ {\rho u^2} \right]_\theta = \left[ {\rho u^2} \right]_{\theta = 0}$, based on analyses of Ulysses observations^67^. We note that the assumption of latitudinal invariance of SW dynamic pressure does not significantly affect our results. The most important factor that affects the timing for SW propagation to the HTS is the SW speed measured at 1 au, while the SW density acts as a less effective, higher-order factor in determining the mass-loading of the SW from 1 au to the HTS. We include an uncertainty for SW density in our analysis, as described further below, but it does not contribute significantly to the uncertainties of the distance results.

We note that IPS-derived SW speeds show an abrupt increase in the southern hemisphere in CR 2,156 compared to CR 2,154 and 2,155. This behaviour appears to be caused by an emission of fast SW from a large coronal hole at mid-latitudes in the southern hemisphere in late 2014 as seen in the Solar Dynamics Observatory (SDO) Atmospheric Imaging Assembly (AIA)/Helioseismic and Magnetic Imager (HMI) observations (Fig. 1f) (https://sdo.gsfc.nasa.gov/data/aiahmi/). This coronal hole is persistent over multiple CRs to early 2015, indicating that the fast SW speeds in the southern hemisphere in CR 2,156 are important to include in our analysis. Because fast SW streams will interact with slow streams preceding them in time, and since our model cannot simulate the fast–slow SW speed interaction, we give larger weighting to SW speeds in CR 2,156 when calculating the weighted average in Fig. 1e (25% for CR 2,154, 25% for CR 2,155 and 50% for CR 2,156). The weighted standard deviation of the average, shown in grey in Fig. 1e, indicates that a relative uncertainty of 15% applied to SW speeds at all latitudes is sufficient to capture the potential uncertainties in our model. If we were to use SW speeds from CR 2,156 only, our HTS and HP distances would move slightly outwards at roughly 0 to −45° latitudes, but the time at which we begin the SW propagation at 1 au would be roughly 0.05 years after that used in our analysis. Ultimately, this would move our heliosphere boundaries only by a few au, and thus not enough to make a statistically significant difference.

The temperature of SW protons, if just solved using equations (2)–(6), yield values below 1,000 K at the HTS. However, Voyager 2 observations clearly show that the SW proton temperature does not decrease adiabatically with distance from the Sun and slightly increases with distance beyond roughly 20 au from the Sun^68^. The reason for this non-adiabatic heating has been studied in detail in the past and is probably due to turbulent heating by waves excited by interstellar PUI injection^45,69–74^. While it is beyond the scope of our analysis to include a turbulent heating source term for SW protons in equations (2)–(6), we can put a lower limit on the SW proton temperature that is roughly consistent with Voyager observations. Thus, when solving the transport of SW proton pressure, we force their temperature to always be ≥10^4^ K. This assumption does not significantly affect our results, however, since the interstellar H and He PUIs dominate the internal pressure of SW in the outer heliosphere. We note that New Horizons’ SWAP observations show the H^+^ PUI temperature is roughly 4 × 10^6^ K at 30 au from the Sun in late 2014 (ref. ^75^), which is close to our model prediction of 4.0 × 10^6^ K at the same distance. This does not necessarily suggest our model is consistent with SWAP at other times or distances from the Sun, because SWAP observations show PUIs experience non-adiabatic heating from a physical process that is not yet fully understood.

The total thermal pressure $p = p_e + \mathop {\sum }\limits_{i = 1}^4 p_i$ includes the pressure of electrons and all ion components. We assume quasi-neutrality is maintained throughout the system. The temperature of electrons in the outer heliosphere is not well known, but there is reason to believe they contain non-negligible suprathermal distributions. Electrons may be substantially heated at interplanetary shocks, maintaining high internal energies compared to the thermal SW protons^76–78^. Therefore, we assume that electron temperatures are ten times higher than the SW protons.

The interstellar neutral H density, *n*_H_, is extracted from a global, three-dimensional steady-state simulation of the heliosphere based on the methodology in Zirnstein et al. (ref. ^79^). The simulation boundary conditions at 1 au are similar to the previous work, but the interstellar neutral H density was increased such that the H density near the upwind HTS is consistent with recent measurements from New Horizons’ SWAP^80^. Interstellar neutral H distribution is assumed to be Maxwellian moving at a bulk speed of *u*_H_ = 22 km s^−1^ and their inflow direction is (252°.2, 9°)^81,82^.

The interstellar neutral He density, *n*_He_ (*r*, *θ*), is calculated analytically for a cold gas^8,83^, such that

12 $$\begin{array}{*{20}{l}} {n_{{\mathrm{He}}}\left( {r,\theta } \right)} \hfill & = \hfill & {n_{{\mathrm{He}},\infty }\mathop {\sum }\limits_{j = 1}^2 \frac{{p_j^2{{{\mathrm{exp}}}}\left( { - \lambda _{{\mathrm{He}}}u_{{\mathrm{He}},\infty }\theta _j/\left| {p_j} \right|} \right)}}{{u_{He,\infty }^2r{{{\mathrm{sin}}}}\theta \left[ {r^2{{{\mathrm{sin}}}}^2\theta - 2rr_p\left( 0 \right)\left( {1 - {{{\mathrm{cos}}}}\theta } \right)} \right]^{1/2}}},} \hfill \\ {r_p\left( 0 \right)} \hfill & = \hfill & {2GM\left(\, {\mu - 1} \right)/u_{{\mathrm{He}},\infty }^2,} \hfill \\ {p_j} \hfill & = \hfill & {\frac{1}{2}u_{{\mathrm{He}},\infty }\left\{ {r{{{\mathrm{sin}}}}\theta \pm \left[ {r^2{{{\mathrm{sin}}}}^2\theta - 2rr_p\left( 0 \right)\left( {1 - {{{\mathrm{cos}}}}\theta } \right)} \right]^{1/2}} \right\},} \hfill \end{array}$$

where $n_{{\mathrm{He}},\infty } = 0.015$ cm^−3^ is the interstellar neutral He density far from the Sun and $\lambda _{{\mathrm{He}}} = 0.5$ au is the size of the He density depletion region due to ionization^84,85^, *G* is the gravitational constant, *M* is the solar mass, $u_{{\mathrm{He}},\infty } = 25.4$ km s^−1^ is the interstellar neutral He speed with inflow direction (255°.7, 5°.1)^86^, *μ* = 0 is the gravity compensation factor due to solar radiation pressure, *θ* is the angle of vector **r** from the neutral He upwind direction and *θ*_*j*_ = *θ* if *p*_*j*_ > 0 and *θ*_*j*_ = 2*π* − *θ* if *p*_*j*_ < 0 (see ref. ^83^ for more details).

By solving equations (2)–(6), the bulk SW speed *u* (*r*) is calculated for each pixel direction in the sky as a function of distance *r* from the Sun in equation (1), therefore allowing us to derive *r*_HTS_. Next, the distance from the HTS to the mean ENA source and HP is calculated. First, we estimate the HTS compression ratio by solving the shock adiabatic equation for a perpendicular shock, given as

13 $$\begin{array}{l}2\left( {2 - \gamma } \right)R^2 + \gamma \left[ {2\left( {1 + \beta _{\mathrm{u}}} \right) + \left( {\gamma - 1} \right)\beta _{\mathrm{u}}M_{\mathrm{u}}^2} \right]R - \gamma \left( {\gamma + 1} \right)\beta _{\mathrm{u}}M_{\mathrm{u}}^2 = 0,\\ \beta _{\mathrm{u}} = \frac{{2\mu _0p_{\mathrm{u}}}}{{B_{\mathrm{u}}^2}},\\ M_{\mathrm{u}} = \frac{{u_{\mathrm{u}}}}{{\sqrt {\gamma p_{\mathrm{u}}/\rho _{\mathrm{u}}} }},\end{array}$$

where *R* is the (unique) shock compression ratio, *β*_u_ is the upstream plasma beta and *M*_u_ is the upstream plasma Mach number. We calculate the upstream SW bulk flow speed, *u*_u_, effective thermal pressure, *p*_u_, for all electron+ion components and effective mass density, *ρ*_u_, and magnetic field *B*_u_, from the solution of equations (2)–(6). We note that the effective specific heat ratio *γ* pressure term *γp*_u_ need not assume that the index *γ* = 5/3 for all ion species, since it is possible that interstellar PUIs behave non-adiabatically due to their unique occupation of phase space and behaviour in the SW^75^. To allow for this possibility, we assumed that $\gamma _{{\mathrm{SW}}} = \gamma _{{\mathrm{PUI}}} = \gamma = 5/3$ for all particles but introduce a relative uncertainty that accounts for the possibility that *γ* might range between 1.33 and 2.0 (Calculation and propagation of uncertainties section). Thus, the effective specific ratio upstream of the HTS is

14 $$\gamma = \frac{{\gamma _{{\mathrm{SW}}}}}{{p_{\mathrm{u}}}}\left( {p_{\mathrm{e}} + p_{{\mathrm{SWH}}^ + } + p_{{\mathrm{SWHe}}^{ + + }}} \right) + \frac{{\gamma _{{\mathrm{PUI}}}}}{{p_{\mathrm{u}}}}\left( {p_{{\mathrm{PUIH}}^ + } + p_{{\mathrm{PUIHe}}^ + }} \right).$$

The final step before calculating the mean ENA source and HP distances involves calculating the fast magnetosonic wave speed in the IHS, which we assume is the dominant wave speed in the IHS. The effective pressure term downstream of the HTS, *p*_d_, is readily calculated from the Rankine–Hugoniot jump conditions for a perpendicular shock as a function of the upstream plasma properties^87^. We also include a contribution of pressure from anomalous cosmic rays (ACRs) that may be on the order of 30% of the total pressure^18,88,89^. Thus, the total effective plasma pressure downstream of the HTS is modified to be $p_{{\mathrm{d}},{\mathrm{tot}}} = p_{\mathrm{d}}/\left( {1 - f_{{\mathrm{ACR}}}} \right)$, where *f*_ACR_ = 0.3. The fast magnetosonic wave speed is then calculated as

15 $$u_{\mathrm{w}} = \sqrt {\frac{{\gamma p_{{\mathrm{d}},{\mathrm{tot}}}}}{{\rho _{\mathrm{d}}}} + \frac{{B_{\mathrm{d}}^2}}{{\mu _0\rho _{\mathrm{d}}}}} ,$$

where *B*_d_ = *B*_u_*R*.

The IHS plasma flow speed immediately downstream of the HTS is derived using the shock compression ratio from equation (13), such that $u_{{\mathrm{d}},0} = u_{\mathrm{u}}/R$, as well as the fast magnetosonic wave speed using the downstream plasma pressure. The downstream flow speed and wave speed are used to simultaneously calculate the radial distance from the HTS through the IHS at which the mean ENA source and HP are located. This is done by performing an iterative, binary search for the optimal solution for the position of the HP that, using the previously derived IHS flow and wave speeds, yields the correct time delay observed by IBEX. The search behaves as:

1. Define an initial search range of ${\Delta}r_{{\mathrm{HP}},{\mathrm{min}}}^i < {\Delta}r_{{\mathrm{HP}}}^i < {\Delta}r_{{\mathrm{HP}},{\mathrm{max}}}^i$ (where *i* represents the step iteration), assuming ${\Delta}r_{{\mathrm{HP}},{\mathrm{min}}}^i = 2$ au and ${\Delta}r_{{\mathrm{HP}},{\mathrm{max}}}^i = 70$ au. The distance to the HP from the HTS is assumed to be in the middle of the range, that is, ${\Delta}r_{{\mathrm{HP}}}^i = \left( {{\Delta}r_{{\mathrm{HP}},{\mathrm{min}}}^i + {\Delta}r_{{\mathrm{HP}},{\mathrm{max}}}^i} \right)/2$.
2. Calculate time Δ*t*_1_ it takes for the forward-propagating wave at speed $u_{{\mathrm{fw}}}\left( r \right) = u_{\mathrm{w}} + u_{\mathrm{a}}\left( r \right)$ to travel from *r*_HTS_ to *r*_HP_, where *u*_w_ is the fast magnetosonic wave speed and *u*_a_(*r*) is the advecting flow speed (see details below).
3. Propagate the advecting flow at speed *u*_a_(*r*) for the same amount of time Δ*t*_1_ through the IHS.
4. Propagate the advecting flow and backward-propagating reflected wave (moving at speed $u_{{\mathrm{rw}}}\left( r \right) = u_{\mathrm{w}} - u_{\mathrm{a}}\left( r \right)$) simultaneously over time until they cross each other and their difference in position ${\Delta}r_{{\mathrm{af}}} - {\Delta}r_{{\mathrm{rw}}} = {\Delta}l_{{\mathrm{ENA}}}/2$ (where we have assumed that ${\Delta}l_{{\mathrm{ENA}}}/2 \cong \left( {r_{{\mathrm{HP}}} - r_{{\mathrm{HTS}}}} \right)/4$ based on simulations^26^), yielding elapsed time Δ*t*_2_. Note that Δ*r*_af_ and Δ*r*_rw_ are radial distances from the HTS.
5. If the HP position *r*_HP_ is the optimal choice, the total elapsed time should equal ${\Delta}t_1 + {\Delta}t_2 = t_{{\mathrm{ENA}}} - t_{{\mathrm{HTS}}} - {\Delta}t_{{\mathrm{ENA}} \to {\mathrm{HTS}}}$, where ${\Delta}t_{{\mathrm{ENA}} \to {\mathrm{HTS}}}$ is the amount of time it takes ENAs at speed *v*_ENA_ to travel from the *r*_ENA_ to *r*_HTS_. The mean ENA source distance from the HTS is then defined as ${\Delta}r_{{\mathrm{ENA}}} = \left( {{\Delta}r_{{\mathrm{af}}} + {\Delta}r_{{\mathrm{rw}}}} \right)/2$, that is, the middle of the intersection of the advecting flow and reflected wave.
6. Compare total elapsed time from Step 5 (${\Delta}t_1 + {\Delta}t_2$) to the time based on IBEX observations ($t_{{\mathrm{ENA}}} - t_{{\mathrm{HTS}}} - {\Delta}t_{{\mathrm{ENA}} \to {\mathrm{HTS}}}$). If the elapsed time from Step 5 is less than the observed time, update the search range for ${\Delta}r_{{\mathrm{HP}}}^{i + 1}$ to be $\left( {{\Delta}r_{{\mathrm{HP}},{\mathrm{min}}}^{i + 1},{\Delta}r_{{\mathrm{HP}},{\mathrm{max}}}^{i + 1}} \right) = ({\Delta}r_{{\mathrm{HP}}}^i,{\Delta}r_{{\mathrm{HP}},{\mathrm{max}}}^i)$. If the elapsed time from Step 5 is greater than the observed time, update the search range for ${\Delta}r_{{\mathrm{HP}}}^{i + 1}$ to be $\left( {{\Delta}r_{{\mathrm{HP}},{\mathrm{min}}}^{i + 1},{\Delta}r_{{\mathrm{HP}},{\mathrm{max}}}^{i + 1}} \right) = ({\Delta}r_{{\mathrm{HP}},{\mathrm{min}}}^i,{\Delta}r_{{\mathrm{HP}}}^i)$. The new estimate for the distance of the HP from the HTS is ${\Delta}r_{{\mathrm{HP}}}^{i + 1} = \left( {{\Delta}r_{{\mathrm{HP}},{\mathrm{min}}}^{i + 1} + {\Delta}r_{{\mathrm{HP}},{\mathrm{max}}}^{i + 1}} \right)/2$.

Steps 2–6 are iteratively repeated until the optimal choice for Δ*r*_HP_ (and thus Δ*r*_ENA_) is found with an accuracy of <0.5 au.

We note that this process computes a radial distance from the HTS with an estimation for IHS plasma flow deflection away from the radial vector. There are no direct observations of IHS plasma flow deflection except for measurements from the Voyager spacecraft over two directions in the sky, which may not be applicable over all directions in our analysis. From global, steady-state simulations, we expect that the plasma flow is slowed near the IHS stagnation point and deflected away from it, although the existence and location of a stagnation point depends on asymmetries induced by the interstellar magnetic field, time-dependent solar cycle effects and corotating interaction regions, and instabilities developing near the HP^32,90–95^.

We first approximate the amount of flow deflection with help from the global heliosphere simulation^79^. We calculate the average flow deflection angle in the IHS plasma as a function of direction in the sky from the simulation, weighted by the 4.3 keV ENA source in the IHS (see ref. ^79^, section 2.2). From the simulation, we find minimal deflection (roughly 0°) near the simulated stagnation point located near ecliptic (267°, −4°), and the deflection angle increases to a maximum of roughly 45° at an angle of roughly 40° away from the stagnation point, nearly symmetric in longitude and latitude. However, the true IHS stagnation point is probably roughly 30° below the nose as determined from IBEX and Voyager observations^47^. Therefore, we use the information from the simulation but modify it to better match these and other observations. We define a function such that the flow deflection angle is zero at (255°, −27°), increases proportional to $\sqrt \varphi$ (where *φ* is the angular separation of the pixel from the stagnation point) and maximizes as 45° at *φ* = 40°. This indicates a decrease of the radial plasma flow speed *u*_a_ (*r*) by a factor of $\cos 45^\circ = 0.7$. Next, we incorporate information from Voyager observations. While Voyager 1 observations indicate slowing can be as large as roughly 50% halfway through the IHS^96^, Voyager 2 observations show less slowing (roughly 25%)^97^ and recent analyses suggest radial plasma flow velocities derived from Voyager 1 energetic particle measurements may be inaccurate^97,98^. Moreover, these observations at Voyager 1 and 2 are probably coupled to time-dependent, solar cycle effects that are nearly impossible to predict for our analysis^95^. Thus, our analysis can only include a rough approximation of this effect. We approximate the IHS plasma flow speed as a function of distance *r* from the HTS according to

16 $$u_{\mathrm{a}}\left( r \right) = u_{{\mathrm{d}},0} \times \left[ {1 - \left( {\frac{r}{{{\Delta}r_{{\mathrm{HP}}}/2}}{{{\mathrm{{\Gamma}}}}}} \right)^2} \right] \times \frac{1}{2}\left[ {1 - {\mathrm{tanh}}\left( {r - 0.95{\Delta}r_{{\mathrm{HP}}}} \right)} \right],$$

where *u*_d,0_ is the initial downstream flow speed, the second term introduces slowing where Γ = 0.5, and the final term requires the flow to reach 0 at the HP for any arbitrary value of Γ. For the nominal value of Γ = 0.5, equation (16) requires that the radial flow speed decreases to 75% halfway through the IHS, similar to Voyager 2 measurements, and drops more quickly closer to the HP.

The distances *r*_HTS_, Δ*r*_ENA_ and Δ*r*_HP_ are solved as a function of ENA speed, *v*_ENA_, and must be integrated over each IBEX ESA energy passband. Because the IBEX-Hi ESA passbands cover a relatively wide range of ENA energies with a full-width at half-maximum of roughly 60% (ref. ^33^), these results must be repeated for a range of ENA energies over ESA passbands 4–6 and weighted by the instrument energy-dependent response functions and ENA flux spectra. Therefore, we solve for *r*_HTS_, Δ*r*_ENA_ and Δ*r*_HP_ over a range of ENA speeds and average the results as

17 $$\begin{array}{l}\left\langle {r_{{\mathrm{HTS}}}} \right\rangle = \frac{{\mathop {\smallint }\nolimits_{v_{{\mathrm{min}}}}^{v_{{\mathrm{max}}}} r_{{\mathrm{HTS}}}\left( v \right)W_{{\mathrm{HTS}}}\left( v \right){\mathrm{d}}v}}{{\mathop {\smallint }\nolimits_{v_{{\mathrm{min}}}}^{v_{{\mathrm{max}}}} W_{{\mathrm{HTS}}}\left( v \right){\mathrm{d}}v}},\\ W_{{\mathrm{HTS}}}\left( v \right) = R\left( v \right)v^{ - \eta _{{\mathrm{HTS}}}},\end{array}$$

18 $$\left\langle {{\Delta}r_{{\mathrm{ENA}}}} \right\rangle = \frac{{\mathop {\smallint }\nolimits_{v_{{\mathrm{min}}}}^{v_{{\mathrm{max}}}} {\Delta}r_{{\mathrm{ENA}}}\left( v \right)W_{{\mathrm{ENA}}}\left( v \right){\mathrm{d}}v}}{{\mathop {\smallint }\nolimits_{v_{{\mathrm{min}}}}^{v_{{\mathrm{max}}}} W_{{\mathrm{ENA}}}\left( v \right){\mathrm{d}}v}},$$

19 $$\begin{array}{l}\left\langle {{\Delta}r_{{\mathrm{HP}}}} \right\rangle = \frac{{\mathop {\smallint }\nolimits_{v_{{\mathrm{min}}}}^{v_{{\mathrm{max}}}} {\Delta}r_{{\mathrm{HP}}}\left( v \right)W_{{\mathrm{ENA}}}\left( v \right){\mathrm{d}}v}}{{\mathop {\smallint }\nolimits_{v_{{\mathrm{min}}}}^{v_{{\mathrm{max}}}} W_{{\mathrm{ENA}}}\left( v \right){\mathrm{d}}v}},\\ W_{{\mathrm{ENA}}}\left( v \right) = R\left( v \right)v^{ - \eta _{{\mathrm{ENA}}}},\end{array}$$

The weights *W*_HTS_ and *W*_ENA_ are calculated as a function of the IBEX ESA energy response function *R*(*v*)^46,99^ and the observed GDF ENA spectral indices *η*_HTS_ and *η*_ENA_ measured by IBEX at times *t*_HTS_ and *t*_ENA_, respectively. Because the observations of *t*_HTS_ and *t*_ENA_ are made at different times, the ENA spectral index is different for the HTS, mean ENA source and HP distance results (note that we use the same weight for Δ*r*_ENA_ and Δ*r*_HP_ because the same observation time is used to derive them). We estimate *η*_HTS_ and *η*_ENA_ as a function of longitude, latitude and time using results from Swaczyna et al. (ref. ^38^), who performed a spherical harmonic decomposition of the IBEX GDF observations after separating out the ribbon and provided full sky maps of the GDF at all IBEX-Hi energies. We use Compton–Getting and survival-probability corrected GDF results derived by their analysis and compute the ENA spectral indices between ESA 3–5 and ESA 4–6 as a function of longitude, latitude and time to estimate *η*_HTS_ and *η*_ENA_ in our results. Spectral indices between ESA 3–5 are used for the distance calculations for ESA 4, and the spectral indices between ESA 4–6 are used for the distance calculations for ESA 5 and 6. The derived spectral indices are interpolated in time at *t*_HTS_ and *t*_ENA_ for each pixel in the sky and used in equations (17)–(19).

The results for $\left\langle {r_{{\mathrm{HTS}}}} \right\rangle$, $\left\langle {{\Delta}r_{{\mathrm{ENA}}}} \right\rangle$ and $\left\langle {{\Delta}r_{{\mathrm{HP}}}} \right\rangle$ are obtained for each ESA 4–6 after integrating equations (17)–(19). Then, we combine the results over energy by averaging the distances with weights determined by the propagated variances. Their corresponding uncertainties are calculated by the propagation of uncertainties of multiple variables used in the analysis (next section).

### Calculation and propagation of uncertainties

Our analysis includes multiple sources of uncertainties and propagates the uncertainties when calculating the distances to the HTS, mean ENA source and HP. The parameters with uncertainties are listed below:

1. IBEX ENA fluxes, *J*_ENA_. We propagate the statistical uncertainties of the IBEX ENA fluxes through the analysis. The relative uncertainties are typically a few percent and therefore do not contribute significantly to most of the results.
2. Initial ENA response time, *t*_HTS_. The uncertainty of the initial ENA response time is propagated through the calculation of the heliospheric boundary distances. This uncertainty is partly composed of the propagated ENA flux uncertainties. Also, the location of the HTS based on the point of local minimum in ENA flux right before the sharp rise is not well constrained due to the potentially substantial fluctuation of ENA fluxes over time unrelated to the large SW pressure increase in late 2014. This variability, examples of which are visible in Fig. 3a,e,g, is not accounted for in the simulation and therefore is used as an estimate of uncertainty in our analysis. This fluctuations before the pressure increase may be realistic effects of the outer heliosphere, or potentially due to imperfect Compton–Getting corrections or background subtractions within ESA 5 and 6 (refs. ^33,100^). Regardless of the origin, we attempt to account for this uncertainty by (1) calculating the standard deviation in IBEX ENA flux over a 1-year period before *t*_HTS_, that is, *s*_*J*_, (2) adding *s*_*J*_ to the flux at the initial response time, that is $J\left( {t_{{\mathrm{HTS}}}} \right) + s_J$, (3) finding the point in time after *t*_HTS_ when the observed flux $J = J\left( {t_{{\mathrm{HTS}}}} \right) + s_J = J\left( {t^ \ast } \right)$ and (4) calculating the difference $t^ \ast - t_{{\mathrm{HTS}}}$. This difference is added in quadrature to the other propagated uncertainties. This uncertainty represents the largest uncertainty in many pixels of the sky.
3. Mean ENA response time, *t*_ENA_. The uncertainty of the mean ENA response time is propagated through the calculation of the heliospheric boundary distances. This uncertainty is primarily composed of the propagated ENA flux uncertainties. While variability in ENA flux exists before the large response of ENAs to the SW pressure event may affect the initial ENA response time significantly, the same may occur with the distance to the HP, but changes to the HP occur more slowly over time and it is expected that wave reflection from the HP happens before the boundary moves outwards by any noticeable distance. The uncertainties in the mean ENA response time are small due to the smooth gradient in ENA flux and are largely due to uncertainties in the IBEX data.
4. SW pressure increase start time. The start time of the SW pressure increase at 1 au is assumed to be near the time of peak gradient in the SW pressure. Specifically, we choose to average the median times of the three CRs available in IPS observations surrounding the peak gradient in SW pressure, that is, CR 2,154–2,156, with double weighting for CR 2,156 due to the abrupt rise in SW speed from a coronal hole in the southern hemisphere. The weight-averaged start time is calculated to be 2014.78. We include a 1-sigma uncertainty of one CR (27.3 days or roughly 0.075 years) in the start time and add it in quadrature to the initial and mean ENA response time uncertainties before calculating the heliosphere boundary distances.
5. IPS SW speed, *u*_0_, and density, *n*_0_. The absolute accuracy of SW speeds derived from IPS observations is difficult to quantify at high latitudes, especially without in situ observations from Ulysses that were available before 2009. Sokół et al. (ref. ^101^) and Tokumaru et al. (ref. ^42^) estimated the uncertainty in IPS-derived SW speeds by calculating the relative variance between IPS and Ulysses observations during polar orbits, yielding a relative error of roughly 10% for speed and roughly 20–30% for density. Our analysis uses SW speeds derived from IPS observations during CR 2,154–2,156, which approximately overlap the middle of the rise in SW dynamic pressure in late 2014. While this choice appeared sufficient for our purposes, and we attempted to account for all potential uncertainties in our analysis, we cannot definitively claim that averaging over these three CRs is the best choice of SW speed to use in our analysis. Second, IPS-derived SW speeds at low latitudes after 2009 are systematically higher by roughly 50–100 km s^−1^ than speeds in the OMNI database observed in situ by spacecraft, the reason for which is not clear but appears to be caused by the evolving relationship between the SW speed and density fluctuations in different solar cycles^42,65^. Because there was agreement between IPS and both OMNI and Ulysses measurements before 2009 (ref. ^101^), it is assumed that any difference between IPS and OMNI now is systematic over all latitudes and thus the IPS-derived SW speeds can be shifted by a common value at all latitudes to match OMNI data^42^. However, it is not clear how accurate this approach is without in situ measurements at high latitudes. Finally, it is important to note that IPS-derived SW speeds in CR 2,156 are higher at heliolatitudes roughly 0 to −45° compared to other latitudes, which approximately overlaps the region in the sky where the HTS and HP distances derived in our analysis are closest to the Sun. If we were to use SW speeds from CR 2,156 only, our HTS and HP distances would move slightly outwards at roughly 0 to −45° latitudes, but the time at which we begin the SW propagation at 1 au would be roughly 0.05 year after that used in our analysis. Ultimately, this would move our heliosphere boundaries only by a few au, and thus not enough to make a statistically significant difference. On the basis of this analysis, and the uncertainty associated with needing to shift IPS-derived SW speeds to match OMNI, in our analysis we assume a relative error of 15% for IPS-derived SW speeds and 25% for IPS-derived SW density and propagate their uncertainties through the analysis.
6. Interstellar neutral H density, *n*_H_, and charge exchange cross section, *σ*. Swaczyna et al. (ref. ^80^) recently updated the interstellar neutral H density within the outer heliosphere, yielding 0.127 ± 0.015 cm^−3^ at the upwind HTS. This is roughly 40% higher compared to previous work, which is obtained from the first outer heliosphere measurements of interstellar H^+^ PUIs by New Horizons SWAP that provided a better estimation of the parent neutral H density. The uncertainty of *n*_H_ obtained in that study includes an estimated uncertainty of the charge exchange cross section of the order of 10%. Therefore, to avoid double counting of the uncertainty of the cross section, we only include the combined uncertainty of interstellar neutral H density from the Swaczyna et al. analysis.
7. SW proton temperature ‘floor’. We noted that the SW proton temperature solved using equations (2)–(6) in the supersonic SW yield unrealistically low values without including turbulent heating source terms. To account for this, we force the SW proton temperature to be at least 10^4^ K at each step in the solution. Clearly, there is significant uncertainty in this approach; therefore, we assume a relative uncertainty of 100% of this parameter and propagate it through the analysis.
8. SW electron temperature, *T*_e_. The temperature of electrons in the supersonic SW is assumed to be ten times higher than the SW protons, which is an assumption based largely on extensive theoretical calculations^76–78,102^. Therefore, we assume a relative uncertainty of 100% (that is, ranging between 0 and 20 times the SW proton temperature) and propagate it through the analysis.
9. Specific heat ratio of PUIs, *γ*. Due to the non-thermal distribution of PUIs and their preferential nature of heating at shocks in the outer heliosphere, it is not clear what the specific heat ratio of PUIs is near the HTS. For simplicity, we assume *γ* = 5/3. New Horizons’ SWAP observations of non-adiabatic PUI heating in the outer heliosphere show that the ‘cooling index’ of PUIs, *α*, which is related to the specific heat ratio as $\alpha = 1/\left( {\gamma - 1} \right)$, is roughly 2.1 halfway to the HTS and may increase to roughly 2.9 at the HTS^75^. Because this is the only direct evidence of the specific heat capacity of PUIs, we assume a relative uncertainty of 20% for *γ*, such that within 1-sigma of uncertainty, *γ* may be between 1.33 and 2.0 (or *α* varies between 3 and 1, respectively).
10. HTS compression ratio, *R*. The kinetic nature of particle heating and acceleration at the HTS probably means that our use of the single-fluid, ideal shock adiabatic equation to derive the HTS compression has some level of uncertainty. While the compression ratio derived using equation (13) yields values that appear consistent with measurements from Voyager 2 (ref. ^103^) and predictions from particle-in-cell simulations^104^, we include a 1-sigma relative uncertainty of 10% for the HTS compression ratio in our analysis.
11. IHS plasma flow speed, *u*_d_. Downstream of the HTS, the plasma flows through the IHS and is deflected away from the radial vector and slowed by compression or deflection near the HP. Considering the substantial differences in Voyager 1 and 2 observations (or differences in interpretations of the data), and what little is known about the global IHS plasma flow, we introduce an uncertainty of the flow slowing factor Γ = 0.5 in an amount of $\sigma _{{{\mathrm{{\Gamma}}}}} = 1/\sqrt 2 - {{{\mathrm{{\Gamma}}}}}$, such that the radial plasma speed halfway through the IHS may be between 75 and 50% slower than its initial speed at the HTS.
12. ACR pressure contribution in the IHS, *p*_ACR_. In our analysis, we assume that ACRs contain 30% of the total effective thermal pressure of the IHS plasma, based on a recent analysis of Voyager and IBEX observations^18^. We assume a relative uncertainty of 33% of this parameter and propagate it through the analysis.
13. ENA source region thickness, Δ*l*_ENA_. To determine the optimal position of the HP, we must assume a distance between the backwards-propagating reflected wave and forwards-propagating advecting flow that coincides with the mean ENA response time. Based on simulation results of Zirnstein et al. (ref. ^26^), the half-width of the 4.3 keV ENA source thickness Δ*l*_ENA_ is approximately 25% of the distance from the HTS to the HP over most directions of the sky used in our analysis. Because we assume that the overlap between the wave and advecting flow must be approximately Δ*l*_ENA_/2 to coincide with the mean ENA response time, *t*_ENA_, our calculation of $\left\langle {{\Delta}r_{{\mathrm{ENA}}}} \right\rangle$ and $\left\langle {{\Delta}r_{{\mathrm{HP}}}} \right\rangle$ relies strongly on this assumption. Therefore, we assume an uncertainty of 100% for this parameter, such that the overlap region might be anywhere between 0 and twice the half-width of the ENA source region and that it cannot be greater than half of the total IHS thickness.

The uncertainties listed above are propagated through each step of the analysis. This is performed by manually varying the values of each parameter by its 1-sigma uncertainty, recalculating the desired variable with the perturbed parameter and adding the deviations of the results in quadrature to estimate the final propagated uncertainty. For example, when calculating $\left\langle {{\Delta}r_{{\mathrm{ENA}}}} \right\rangle$, its uncertainty is calculated as

20 $$\sigma _{r,{\mathrm{ENA}}} = \sqrt {\mathop {\sum }\limits_{j = 1}^N \left( {\left\langle {{\Delta}r_{{\mathrm{ENA}}}} \right\rangle - \left. {\left\langle {{\Delta}r_{{\mathrm{ENA}}}} \right\rangle } \right|_j} \right)^2} {{{\mathrm{,}}}}$$

where index *j* represents the parameter whose value was increased by its 1-sigma uncertainty before recalculating $\left. {\left\langle {{\Delta}r_{{\mathrm{ENA}}}} \right\rangle } \right|_j$. We note that this method assumes all parameters are independent.

Averaging results with uncertainties, such as angular smoothing over multiple pixels, is performed by weighting values by their inverse variances and calculating the uncertainty of the average. As an example, for arbitrary variable *g* with uncertainty *σ*_*g*_, its weighted average, $\left\langle g \right\rangle$, is calculated as

21 $$\left\langle g \right\rangle = \frac{{\mathop {\sum }\nolimits_{j = 1}^N g_j/\sigma _{g,\,j}^2}}{{\mathop {\sum }\nolimits_{j = 1}^N 1/\sigma _{g,\,j}^2}},$$

The uncertainty, $\sigma_{\left\langle g \right\rangle}$, is calculated from one of the following equations: the propagated uncertainty

22 $$\sigma _g^p = \sqrt {\frac{1}{{\mathop {\sum }\nolimits_{j = 1}^N 1/\sigma _{g,\,j}^2}}} ,$$

or the statistical uncertainty

23 $$\sigma _g^s = \sqrt {\frac{{N_{{\mathrm{eff}}}}}{{N_{{\mathrm{eff}}} - 1}}\frac{{\mathop {\sum }\nolimits_{j = 1}^N \left( {g_j - {\left\langle g \right\rangle}} \right)^2/\sigma _{g,\,j}^2}}{{\mathop {\sum }\nolimits_{j = 1}^N 1/\sigma _{g,\,j}^2}}} ,$$

$$N_{{\mathrm{eff}}} = \frac{{\left( {\mathop {\sum }\nolimits_{j = 1}^N 1/\sigma _{g,\,j}^2} \right)^2}}{{\mathop {\sum }\nolimits_{j = 1}^N 1/\sigma _{g,\,j}^4}},$$

where *N*_eff_ is the effective number of measurements. In the case where the uncertainties of all points are similar, *N*_eff_ approaches the actual number of points, *N*. Equation (21) is used to calculate the weighted average of any variables throughout the analysis. The uncertainty of the weighted average is chosen from either equation (22) or equation (23), whichever is larger.

## Data Availability

The results reported in the study shown in Fig. 4 and Fig. 5a–f are publicly available to download. Source data are provided with this paper.

## Source data

Source Data Fig. 4a,c Initial ENA response times for ESA 4 and their uncertainties from Fig. 4.
Source Data Fig. 4e,g Initial ENA response times for ESA 5 and their uncertainties from Fig. 4.
Source Data Fig. 4i,k Initial ENA response times for ESA 6 and their uncertainties from Fig. 4.
Source Data Fig. 4b,d Mean ENA response times for ESA 4 and their uncertainties from Fig. 4.
Source Data Fig. 4f,h Mean ENA response times for ESA 5 and their uncertainties from Fig. 4.
Source Data Fig. 4j,l Mean ENA response times for ESA 6 and their uncertainties from Fig. 4.
Source Data Fig. 5a,d Distances to the HTS and their uncertainties from Fig. 5.
Source Data Fig. 5b,e Distances to the mean ENA source and their uncertainties from Fig. 5.
Source Data Fig. 5c,f Distances to the HP and their uncertainties from Fig. 5.

## Code availability

Code algorithms used to produce results of this study are available from the corresponding author upon reasonable request.

## References

[1] Parker EN (1961). The stellar-wind regions. Astrophys. J..

[2] McComas DJ (2012). The heliosphere’s interstellar interaction: no bow shock. Science.

[3] Zank GP (2013). Heliospheric structure: the bow wave and the hydrogen wall. Astrophys. J..

[4] Gurnett DA, Kurth WS, Burlaga LF, Ness NF (2013). In situ observations of interstellar plasma with Voyager 1. Science.

[5] Stone EC (2013). Voyager 1 observes low-energy galactic cosmic rays in a region depleted of heliospheric ions. Science.

[6] Stone EC, Cummings AC, Heikkila BC, Lal N (2019). Cosmic ray measurements from Voyager 2 as it crossed into interstellar space. Nat. Astron..

[7] Richardson JD, Belcher JW, Garcia-Galindo P, Burlaga LF (2019). Voyager 2 plasma observations of the heliopause and interstellar medium. Nat. Astron..

[8] Blum PW, Fahr HJ (1970). Interaction between interstellar hydrogen and the solar wind. Astron. Astrophys..

[9] Wallis MK (1975). Local interstellar medium. Nature.

[10] Baranov VB, Malama YuG (1993). Model of the solar wind interaction with the local interstellar medium numerical solution of self-consistent problem. J. Geophys. Res..

[11] McComas DJ (2009). IBEX—Interstellar Boundary Explorer. Space Sci. Rev..

[12] McComas DJ (2009). Global observations of the interstellar interaction from the Interstellar Boundary Explorer (IBEX). Science.

[13] Möbius E (2009). Direct observations of interstellar H, He, and O by the Interstellar Boundary Explorer. Science.

[14] Witte M (2004). Kinetic parameters of interstellar neutral helium. Review of results obtained during one solar cycle with the Ulysses/GAS-instrument. Astron. Astrophys..

[15] Wood BE, Müller H-R, Witte M (2015). Revisiting Ulysses observations of interstellar helium. Astrophys. J..

[16] McComas DJ (2020). Solar cycle of imaging the global heliosphere: Interstellar Boundary Explorer (IBEX) observations from 2009–2019. Astrophys. J. Suppl. Ser..

[17] Richardson JD (2017). Pressure pulses at Voyager 2: drivers of interstellar transients?. Astrophys. J..

[18] Rankin JS, McComas DJ, Richardson JD, Schwadron NA (2019). Heliosheath properties measured from a Voyager 2 to Voyager 1 transient. Astrophys. J..

[19] Burlaga LF (2021). Magnetic fields observed by Voyager 2 in the heliosheath. Astrophys. J..

[20] Stone EC (2005). Voyager 1 explores the termination shock region and the heliosheath beyond. Science.

[21] Stone EC (2008). An asymmetric solar wind termination shock. Nature.

[22] Zirnstein EJ, Dayeh MA, McComas DJ, Sokół JM (2020). Asymmetric structure of the solar wind and heliosphere from IBEX observations. Astrophys. J..

[23] Sokół JM (2021). Breathing of the heliosphere. Astrophys. J..

[24] McComas DJ (2018). Heliosphere responds to a large solar wind intensification: decisive observations from IBEX. Astrophys. J..

[25] McComas DJ (2019). Expanding global features in the outer heliosphere. Astrophys. J..

[26] Zirnstein EJ (2018). Simulation of the solar wind dynamic pressure increase in 2014 and its effect on energetic neutral atom fluxes from the heliosphere. Astrophys. J..

[27] McComas DJ, Rankin JS, Schwadron NA, Swaczyna P (2019). Termination shock measured by Voyagers and IBEX. Astrophys. J..

[28] Reisenfeld, D. B. et al. A three-dimensional map of the heliosphere from IBEX. *Astrophys. J. Suppl. Ser*. **254**, 40 (2021).

[29] Izmodenov V, Malama Y, Ruderman MS (2005). Solar cycle influence on the interaction of the solar wind with Local Interstellar Cloud. Astron. Astrophys..

[30] Washimi H (2011). Realistic and time-varying outer heliospheric modelling. Mon. Not. R. Astron. Soc..

[31] Borovikov SN, Pogorelov NV, Zank GP, Kryukov IA (2008). Consequences of the heliopause instability caused by charge exchange. Astrophys. J..

[32] Borovikov SN, Pogorelov NV (2014). Voyager 1 near the heliopause. Astrophys. J..

[33] McComas DJ (2012). The first three years of IBEX observations and our evolving heliosphere. Astrophys. J. Suppl. Ser..

[34] Forman MA (1970). The Compton-Getting effect for cosmic-ray particles and photons and the Lorentz-invariance of distribution functions. Planet. Space Sci..

[35] McComas DJ (2010). Evolving outer heliosphere: large-scale stability and time variations observed by the Interstellar Boundary Explorer. J. Geophys. Res. Space Phys..

[36] Schwadron NA (2011). Separation of the interstellar boundary explorer ribbon from globally distributed energetic neutral atom flux. Astrophys. J..

[37] Dayeh MA (2019). Variability in the position of the IBEX ribbon over nine years: more observational evidence for a secondary ENA source. Astrophys. J..

[38] Swaczyna P (2022). IBEX ribbon separation using spherical harmonic decomposition of the globally distributed flux. Astrophys. J. Suppl. Ser..

[39] Zirnstein EJ, Heerikhuisen J, McComas DJ (2015). Structure of the Interstellar Boundary Explorer ribbon from secondary charge-exchange at the solar–interstellar interface. Astrophys. J..

[40] Zirnstein EJ (2016). Local interstellar magnetic field determined from the Interstellar Boundary Explorer ribbon. Astrophys. J..

[41] King JH, Papitashvili NE (2005). Solar wind spatial scales in and comparisons of hourly wind and ACE plasma and magnetic field data. J. Geophys. Res. Space Phys..

[42] Tokumaru M, Fujiki K, Kojima M, Iwai K (2021). Global distribution of the solar wind speed reconstructed from improved tomographic analysis of interplanetary scintillation observations between 1985 and 2019. Astrophys. J..

[43] Holzer TE (1972). Interaction of the solar wind with the neutral component of the interstellar gas. J. Geophys. Res..

[44] Pogorelov NV, Bedford MC, Kryukov IA, Zank GP (2016). Pickup ion effect of the solar wind interaction with the local interstellar medium. J. Phys. Conf. Ser..

[45] Zank GP (2018). The pickup ion-mediated solar wind. Astrophys. J..

[46] Funsten HO (2009). The Interstellar Boundary Explorer High Energy (IBEX-Hi) neutral atom imager. Space Sci. Rev..

[47] McComas DJ, Schwadron NA (2014). Plasma flows at Voyager 2 away from the measured suprathermal pressures. Astrophys. J. Lett..

[48] Katushkina O, Izmodenov V, Koutroumpa D, Quémerais E, Jian LK (2019). Unexpected behavior of the solar wind mass flux during solar maxima: two peaks at middle heliolatitudes. Sol. Phys..

[49] Schwadron NA (2009). Comparison of interstellar boundary explorer observations with 3D global heliospheric models. Science.

[50] Pogorelov NV (2011). Interstellar boundary explorer measurements and magnetic field in the vicinity of the heliopause. Astrophys. J..

[51] Izmodenov VV, Alexashov DB (2020). Magnitude and direction of the local interstellar magnetic field inferred from Voyager 1 and 2 interstellar data and global heliospheric model. Astron. Astrophys..

[52] Wang C, Belcher JW (1999). The heliospheric boundary response to large-scale solar wind fluctuations: a gasdynamic model with pickup ions. J. Geophys. Res..

[53] Izmodenov VV, Malama YG, Ruderman MS (2008). Modeling of the outer heliosphere with the realistic solar cycle. Adv. Space Res..

[54] Pogorelov NV (2017). Three-dimensional features of the outer heliosphere due to coupling between the interstellar and heliospheric magnetic field. V. The bow wave, heliospheric boundary layer, instabilities, and magnetic reconnection. Astrophys. J..

[55] Burlaga LF (2019). Magnetic field and particle measurements made by Voyager 2 at and near the heliopause. Nat. Astron..

[56] Washimi H, Tanaka T, Zank GP (2017). Time-varying heliospheric distance to the heliopause. Astrophys. J..

[57] McComas DJ (2018). Interstellar Mapping and Acceleration Probe (IMAP): a new NASA mission. Space Sci. Rev..

[58] Wurz P (2009). IBEX backgrounds and signal-to-noise ratio. Space Sci. Rev..

[59] McComas DJ (2014). IBEX: the first five years (2009–2013). Astrophys. J. Suppl. Ser..

[60] Isenberg, P. A. Interaction of the solar wind with interstellar neutral hydrogen: three-fluid model. *J. Geophys. Res*. **91**, 9965–9972 (1986).

[61] Pauls HL, Zank GP, Williams LL (1995). Interaction of the solar wind with the local interstellar medium. J. Geophys. Res..

[62] Lee MA (2009). Physical processes in the outer heliosphere. Space Sci. Rev..

[63] Swaczyna P, Grzedzielski S, Bzowski M (2014). Assessment of energetic neutral he atom intensities expected from the ibex ribbon. Astrophys. J..

[64] Lindsay BG, Stebbings RF (2005). Charge transfer cross sections for energetic neutral atom data analysis. J. Geophys. Res..

[65] Sokół JM, McComas DJ, Bzowski M, Tokumaru M (2020). Sun-heliosphere observation-based ionization rates model. Astrophys. J..

[66] Bzowski, M. et al. in *Cross-Calibration of Far UV Spectra of Solar System Objects and the Heliosphere* (eds. Quémerais, E. et al.) 67–138 (Springer, 2013).

[67] McComas DJ (2008). Weaker solar wind from the polar coronal holes and the whole Sun. Geophys. Res. Lett..

[68] Richardson JD, Smith CW (2003). The radial temperature profile of the solar wind. Geophys. Res. Lett..

[69] Zank GP, Matthaeus WH, Smith CW (1996). Evolution of turbulent magnetic fluctuation power with heliospheric distance. J. Geophys. Res..

[70] Matthaeus WH, Zank GP, Smith CW, Oughton S (1999). Turbulence, spatial transport, and heating of the solar wind. Phys. Rev. Lett..

[71] Smith CW (2001). Heating of the low-latitude solar wind by dissipation of turbulent magnetic fluctuations. J. Geophys. Res..

[72] Isenberg PA (2005). Turbulence-driven solar wind heating and energization of pickup protons in the outer heliosphere. Astrophys. J..

[73] Adhikari L (2017). II. Transport of nearly incompressible magnetohydrodynamic turbulence from 1 to 75 au. Astrophys. J..

[74] Pine ZB (2020). Solar wind turbulence from 1 to 45 au. IV. Turbulent transport and heating of the solar wind using Voyager observations. Astrophys. J..

[75] McComas DJ (2021). Interstellar pickup ion observations halfway to the termination shock. Astrophys. J. Suppl. Ser..

[76] Chalov SV, Fahr HJ (2013). The role of solar wind electrons at the solar wind termination shock. Mon. Not. R. Astron. Soc..

[77] Chashei IV, Fahr HJ (2014). On solar-wind electron heating at large solar distances. Sol. Phys..

[78] Fahr HJ, Chashei IV, Verscharen D (2014). Traveling solar-wind bulk-velocity fluctuations and their effects on electron heating in the heliosphere. Astron. Astrophys..

[79] Zirnstein EJ, Dayeh MA, Heerikhuisen J, McComas DJ, Swaczyna P (2021). Heliosheath proton distribution in the plasma reference frame. Astrophys. J. Suppl. Ser..

[80] Swaczyna P (2020). Density of neutral hydrogen in the Sun’s interstellar neighborhood. Astrophys. J..

[81] Lallement R (2005). Deflection of the interstellar neutral hydrogen flow across the heliospheric interface. Science.

[82] Lallement, R. et al. The Interstellar H flow: updated analysis of SOHO/SWAN data. In *Proc. IP Conference* Vol. 1216 (Eds. Maksimovic, M. et al.) 555–558 (AIP, 2010).

[83] Thomas GE (1978). The interstellar wind and its influence of the interplanetary environment. Annu. Rev. Earth Planet. Sci..

[84] Gloeckler G (2004). Observations of the helium focusing cone with pickup ions. Astron. Astrophys..

[85] Sokół JM, Kubiak MA, Bzowski M (2019). Interstellar neutral gas species and their pickup ions inside the heliospheric termination shock: the large-scale structures. Astrophys. J..

[86] McComas DJ (2015). Local interstellar medium: six years of direct sampling by *Ibex*. Astrophys. J. Suppl. Ser..

[87] Gurnett, D. A. & Bhattacharjee, A. *Introduction to Plasma Physics* (Cambridge Univ. Press, 2005).

[88] Guo X, Florinski V, Wang C (2018). Effects of anomalous cosmic rays on the structure of the outer heliosphere. Astrophys. J..

[89] Livadiotis G, McComas DJ, Schwadron NA, Funsten HO, Fuselier SA (2013). Pressure of the proton plasma in the inner heliosheath. Astrophys. J..

[90] Zank, G. P. The dynamical heliosphere. In *Proc. AIP Conference* Vol. 471 (Eds. Habbal, S. R. et al.) 783–786 (AIP, 1999).

[91] Florinski V, Zank GP, Pogorelov NV (2005). Heliopause stability in the presence of neutral atoms: Rayleigh-Taylor dispersion analysis and axisymmetric MHD simulations. J. Geophys. Res. Space Phys..

[92] Borovikov SN, Pogorelov NV, Ebert RW (2012). Solar rotation effects on the heliosheath flow near solar minima. Astrophys. J..

[93] Opher M, Drake JF, Velli M, Decker RB, Toth G (2012). Near the boundary of the heliosphere: a flow transition region. Astrophys. J..

[94] Avinash K, Zank GP, Dasgupta B, Bhadoria S (2014). Instability of the heliopause driven by charge exchange interactions. Astrophys. J..

[95] Pogorelov NV (2017). Heliosheath processes and the structure of the heliopause: modeling energetic particles, cosmic rays, and magnetic fields. Space Sci. Rev..

[96] Krimigis SM, Roelof EC, Decker RB, Hill ME (2011). Zero outward flow velocity for plasma in a heliosheath transition layer. Nature.

[97] Richardson JD (2021). Using magnetic flux conservation to determine heliosheath speeds. Astrophys. J..

[98] Cummings AC (2021). No stagnation region before the heliopause at Voyager 1? Inferences from new Voyager 2 results. Astrophys. J..

[99] Schwadron NA (2009). The Interstellar Boundary Explorer Science Operations Center. Space Sci. Rev..

[100] McComas DJ (2017). Seven years of imaging the global heliosphere with IBEX. Astrophys. J. Suppl. Ser..

[101] Sokół JM, Bzowski M, Tokumaru M, Fujiki K, McComas DJ (2013). Heliolatitude and time variations of solar wind structure from in situ measurements and interplanetary scintillation observations. Sol. Phys..

[102] Fahr HJ, Richardson JD, Verscharen D (2015). The electron distribution function downstream of the solar-wind termination shock: where are the hot electrons?. Astron. Astrophys..

[103] Richardson JD, Kasper JC, Wang C, Belcher JW, Lazarus AJ (2008). Cool heliosheath plasma and deceleration of the upstream solar wind at the termination shock. Nature.

[104] Kumar R, Zirnstein EJ, Spitkovsky A (2018). Energy distribution of pickup ions at the solar wind termination shock. Astrophys. J..

